# Detection of serum and salivary IgE and IgG_1_ immunoglobulins specific for diagnosis of food allergy

**DOI:** 10.1371/journal.pone.0214745

**Published:** 2019-04-17

**Authors:** Marília Porto Oliveira Nunes, Maurício Fraga van Tilburg, Eridan Orlando Pereira Tramontina Florean, Maria Izabel Florindo Guedes

**Affiliations:** Laboratory of Biotechnology and Molecular Biology, State University of Ceará, Fortaleza, Ceará, Brazil; Harvard Medical School, UNITED STATES

## Abstract

Given the growing incidence and prevalence of life-threatening food allergies, health concerns have raised new perspectives for *in vivo* and *in vitro* diagnostic methodologies, pointing to saliva as a promising material, already used to diagnose other pathologies. Based on the above considerations, this study aimed to verify the possible use of saliva for the detection of IgE and IgG_1_ in the diagnosis of food allergy. This was a randomized, cross-sectional clinical study with a quantitative approach, developed at a hospital referral center in allergy in the state of Ceará, from January to July 2015. The sample consisted of 36 children of both sexes, aged between 1 and 60 months, with a diagnosis of cow's milk protein allergy (CMPA) by the RAST test. Children hospitalized or under immunosuppressive drugs were excluded from the study. Serum and saliva samples of the participants were collected and subsequently subjected to the indirect immunoenzymatic assay (ELISA) for the detection of specific serum and salivary immunoglobulins for food: corn, papaya, cow's milk, egg white, wheat, soybeans, peanuts, nuts, kiwi, cacao, fish, shrimp, bananas and tomatoes. For comparison of serum and saliva results, the T-test of independent samples and Mann-Whitney were adopted, for samples with normal and non-normal distribution respectively. A confidence interval of 95% was adopted for significant results. It was observed that 100% (n = 36) of the participants presented cow's milk allergy through the indirect ELISA, detecting IgE or IgG_1_ in serum and saliva. When serum IgE and IgG_1_ concentrations were compared, there was no statistical difference (p > 0.05) in 12 of the 14 foods evaluated. The same amount (n = 12) of non-significant differences (p > 0.05) was observed in the comparison of the 14 foods under IgE and IgG_1_ contractions in saliva. In the verification of the average values of IgE present in the serum and saliva of the foods, only cow's milk, fish and papaya showed statistically significant differences (p < 0.05). Of the total food evaluated, only the average levels of IgG_1_ present in serum and saliva showed a significant value (p < 0.05) in banana and tomato. These findings indicate that the detection of IgE and IgG_1_ in saliva proves to be as efficient as in the serum. The use of the salivary technique for use in the diagnosis of food allergy is suggested.

## Introduction

Food allergy (FA) is an adverse health effect due to an immune response that occurs reproducibly after exposure to a given food [1]. Allergies have an impact on the health of the patient, cause expenses for being a selective disease, imposing on their patients certain specific eating behaviors and other expenses with diagnosis and treatment [2].

Thus, FA comes with significant social and economic impact, worrying the scientific community and health professionals. Currently, food allergy is present in more than 220 million people in the world [3]. Therefore, approximately 8% of the infant population and 5% of the adult world population present allergy to at least one food [4].

As it is a larger child population, in recent years new perspectives in the diagnosis for food allergy *in vivo* and *in vitro* have been elucidated, evaluating not only the immunological mechanism with the presence of IgE antibodies, but also the design of the clinical phenotype of food hypersensitivity reactions, thus ensuring the diagnosis of reliable allergy [3].

However, the methods currently available to diagnose FA are still invasive, causing discomfort to the patient, often present high costs, and are restricted to one food per test. In addition, the test considered gold standard in diagnosis, requires hospital environment and trained staff, and may trigger life-threatening reaction, leading to anaphylactic shock in the patient. Moreover, this variety of methods only evaluates the presence of IgE class specific antigen antibodies [5].

In addition to the existence of diagnostic tests through the detection of IgE, other immunoglobulins are also being studied for this purpose. Mention may be made of food antigen-specific IgG_1_ and IgG_4_ panels, as these antibodies have been cited by some authors as a possible alternative for the diagnostic of FA, but currently only IgG_1_ is being further emphasized by the fact that IgG_4_ does not have specific receptors in the cells, which prevents the formation of the clinical reaction of food hypersensitivity [6].

Among the samples investigated for use in the diagnosis of FA [7], saliva has been pointed out by researchers, due to the significant presence of secretory IgA, IgG and IgM immunoglobulins [8] and already have been used for the diagnosis of HIV (human immunodeficiency virus), cancer, tuberculosis and *Helicobacter Pylori* infection [9].

The racional behind the technique used in this study is that some antibody associated with allergy found in the bloodstream can also be found in the saliva, thus allowing them to be identified through immunologycal testing [10,11]. In view of the above stated, this study aimed to verify the possibility of using saliva to detect IgG_1_ for the diagnosis of food allergy.

## Material and methods

### Study participants and sample

This is a case-control study, cross-sectional and quantitative approach, with data collection performed at the food allergy outpatient clinic of a Children's Hospital of reference in Fortaleza / CE and analyzes in the Laboratory of Molecular Biology of the State University of Ceará.

The study comprised of samples from a group of children from 0 to 5 years of age, that attended at the food allergy outpatient clinic, diagnosed with cow's milk protein allergy (CMPA) by the gastro-pediatrician through the RAST [12] test and the anamnesis (Supplementary 1). Only after, those responsible who agreed to participate signed the written informed consent form (WICF) and answered a questionnaire, the collection of serum and saliva were performed. Children hospitalized or taking immunosuppressive drugs were excluded from the study.

For the control group of the study, blood and saliva were collected from healthy children under routine outpatient pediatric care, having the signature of the WICF by their guardians and responses to the food research questionnaire to confirm the absence of allergy to the foods tested.

In order to compose the serum and saliva samples, 36 children, allergic to cow’s milk protein (experimental group), were selected by the inclusion criteria. The ELISA test was performed in 56 children to investigate the presence of IgE and IgG_1_ in serum and saliva samples, firstly, against cow’s milk proteins and posteriorly the other 13 foods, totaling 14 foods studied.

### Ethical and legal aspects

The study was submitted and approved by the Ethics Committee of the State University of Ceará (UECE)–process n° 26108713.6.0000.5534, following all the recommendations of Resolution 466 of the Brazilian National Health Council of December 12, 2012 for research involving human beings.

### Sample preparation

Three milliliters of blood were collected from both groups, stored in heparin-free tubes and centrifuged for 10 minutes at 8,000 x g, for serum withdrawal and storage at -20° C for further analysis. The total saliva was collected in an unstimulated way in the morning, not requiring fasting, through a Swab for collecting saliva (salivette), which was placed in a polypropylene tube and kept on ice. The samples were processed by adapting the Hu et al., (2010) [13] model, where the samples were centrifuged at 8,000 x g for 5 minutes at 4° C. After centrifugation, the supernatants were removed and stored at -20°C until analysis.

### Obtaining food extracts

The extracts were obtained from fresh foods: pasteurized skimmed cow’s milk, ear of corn (*Zea mays*), dehydrated soybeans *(Glycine max)*, peanuts in shell unsalted (*Arachis hypogaea L*.), cashew nut (*Anacardium occidentale*), egg white *(Gallus gallus domesticus)*, papaya *(Carica papaya)*, kiwi *(Actinidia deliciosa)*, fish *(Cynoscion acoupa)*, wheat *(Triticum aestivum)*, *banana (Musa cavendishi)*, tomato *(Solanum lycopersicum)*, cocoa *(Theobroma cacao)*, shrimp *(Litopenaeus vannamei)*.

To obtain the extracts, the foods were triturated with distilled water and then centrifuged at 4° C for 30 minutes at 10,000 x g. The supernatant was removed and mixed with a solution of 10% Acetone (LAMY et al., 2012), diluted 1:3 and incubated for 2h at -20°C. This solution was centrifuged another 2 times at 4°C for 30 minutes with Acetone at 10,000 x g, for pellet formation and diluted with distilled water for quantification by the Bradford method (1976) (CARVALHO et al., 2004), with bovine serum albumin (BSA) as the standard (Table 1) and characterization of the proteins by 15% SDS-PAGE.

**Table 1:** Quantification of total proteins by the method of Bradford (1976).

Food	Amount	Protein concentration (mg/mL)
Pasteurized Skimmed Milk	50mL	1.20
Shrimp*	40g	0.16
Egg White*	50g	0.63
Kiwi*	365g	0.33
Wheat (flour)	60g	15.90
Soybean (PTN)	60g	15.40
Corn*	100g	1.60
Cashew nut	30g	2.50
Peanut	50g	1.70
Cocoa powder	55g	5.60
Fish (yellow hake fillet) *	60g	14.47
Tomato*	590g	0.17
Banana*	250g	0.39
Papaya*	510g	0.14

* Fresh Food

### ELISA Indirect (Enzyme linked immunosorbent assay)

Patients’ serum and saliva were submitted to the indirect ELISA test (Enzyme Linked Immunosorbent Assay) for detection of IgE and IgG_1_, placed on individual plaques. For this test, the plates (U96-POLYSORP-NUNC-IMMUNO PLATE BATCH 016181) of each patient were sensitized with 2μg/well of food (cow’s milk, fish, shrimp, egg, peanut, nut, soy, wheat, corn, papaya, tomato, kiwi, banana and cocoa), diluted in 50 mM sodium carbonate buffer pH 9.6, so that each well was coated with 100 μL of the final solution.

For each food, 4 plates were used, divided in Saliva IgE, Serum IgE, Saliva IgG1 and Serum IgG1, each consisting of duplicated samples, positive, negative and white control. After sensitization with the antigens, the plates were incubated for 18 h at a temperature of 4°C and washed 3 times with PBS-Tween 0.05%, for further blocking with PBS gelatin at 1% and incubated again for 2 hours. After further washing with PBS-Tween 0.05%, each well received serum or saliva from the experimental group diluted 1:100 and incubated for 2 hours at 37° C under gentle shaking. Again the plates were washed 3 times with PBS-Tween 0.05% and added antibodies conjugated to anti-human IgE peroxidase (Sigma A9667) at a dilution of 1:2000 or anti-human IgG1 (Sigma B6775) in 1:1000, incubated under gentle shaking for 1 hour. After washing, the plates received 100 μL/well of tetramethylbenzidine solution (TMB) then the absorbance was measured at 605 nm with an automatic ELISA reader.

For the analysis of the results, the absorbance averages of the blood samples and saliva of the control group were made, which served to establish the cutoff point in the experimental group, calculated and presented as three times the standard deviation of the mean of the negatives [14]. The values found above cutoff were considered positive for food allergy.

### Statistical analysis

The statistical analyzes of this work were carried out using the software R version 3.4.1. Data were expressed as measures of central tendency and dispersion. Also presented error bar graphs with mean and confidence interval of 95%. Normality of the variables was tested using the Kolmogorov-Smirnov test and homogeneity of variances by the Levene test. For the comparison of means, Student’s t-test was used for independent samples, when the data were normal and homogeneous, and the Mann-Whitney test, when the data were non-normal and non-homogeneous. For the comparison between more than two means, when the data were presented normal and homogeneous the test of ANOVA was used, and if not, the Kruskal-Wallis test, which were followed by the Tukey post-test. For the comparison of the mean values of the variables with the reference value, Student’s t-test was used for one sample. The results were considered significant with values of p < 0.05.

## Results

**Fig 1:**
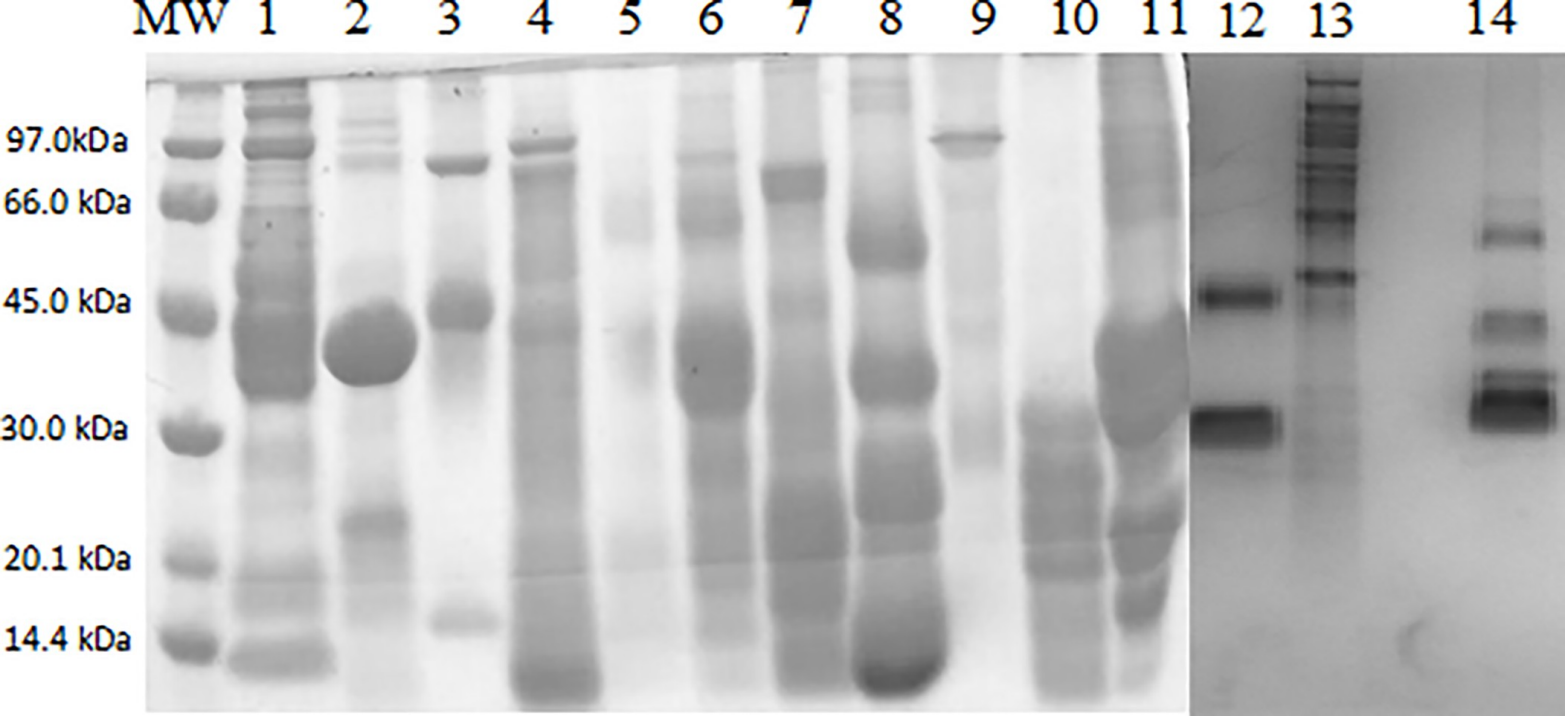
Characterization of total proteins demonstrated in 15% SDS-PAGE stained in Coomassie blue. MW. Molecular Weight, 1. Fish, 2. Shrimp, 3. Clear Egg, 4. Corn, 5. Wheat, 6. Soy, 7. Peanut, 8. Chestnut, 9. Papaya, 10. Kiwi, 11. Milk of cow, 12. Banana, 13. Tomato and 14. Cocoa.

The quantification of the total proteins of the 14 foods in the study was performed by the Bradford method (1976), presented in Table 1, and the electrophoretic profile for protein characterization were obtained by SDS-PAGE, which revealed the migration of protein fractions with an estimated molecular mass in the range of < 14.4 and < 97.0 kDa, which can be observed in Fig 1.

The ELISA test showed a 100% level in both specificity and sensitivity compared to the RAST test. As shown in Table 2, in all the patients that presented allergy to cow’s milk protein, IgE and IgG_1_ was detected in serum and in saliva for the other foods studied, by the ELISA test. On the other hand, no reaction was observed with the control group.

**Table 2:** Distribution of IgE and IgG_1_ present in serum and saliva of allergic children for each food tested by ELISA.

	Serum		Saliva	
	IgE (n)	IgG_1_ (n)	IgE (n)	IgG_1_ (n)
Cow milk	100% (36)	100% (36)	100% (36)	100% (36)
Fish	100% (36)	100% (36)	100% (36)	100% (36)
Shrimp	67% (24)	64% (23)	67% (24)	80% (29)
Egg white	39% (14)	61% (22)	55% (20)	44% (16)
Soy	52% (18)	55% (20)	55% (20)	53% (19)
Wheat	55% (20)	61% (22)	64% (23)	67% (24)
Cashew nut	39% (14)	47% (17)	30% (11)	53% (19)
Peanut	36% (13)	39% (14)	25% (9)	25% (9)
Cocoa	11% (4)	3% (1)	6% (2)	6% (2)
Corn	36% (13)	25% (9)	39% (14)	25% (9)
Papaya	25% (9)	58% (21)	16% (6)	16% (6)
Kiwi	8% (3)	19% (7)	19% (7)	11% (4)
Banana	25% (9)	30% (11)	22% (8)	36% (13)
Tomato	11% (4)	16% (6)	22% (8)	39% (14)

After evaluating the test with cow’s milk protein, it was in the interest of the study to verify if children allergic to cow’s milk protein could present allergy to other foods. The fish, shrimp, egg white, soybean, wheat, peanut, brown, corn, papaya, banana, kiwi, tomato and cocoa proteins were tested in the experimental group, 100% of the sample was also allergic to fish (Table 2). There was a higher prevalence of allergy for ELISA-tested patients in shrimp followed by wheat, egg white, soy, brown, peanut, corn, banana, papaya, tomato, kiwi and cocoa, which presented the lowest percentage of allergic sensitivity, that the animal protein group was the most sensitive when compared to the other groups.

When the means of IgE and IgG_1_ concentrations in serum were compared, all foods had no statistical difference (p> 0.05), except for cow’s milk and fish (p < 0.001; p < 0.010), respectively (Table 3).

**Table 3:** Comparing the average concentration of IgE with the concentration of IgG1 in serum in each food by ELISA test.

Food	Parameter	n	Average (O.D.)	Standard deviation	p
Cow milk	IGE	36	0.430	0.078	< 0.001^£^
	IGG_1_	36	0.339	0.048	
Fish	IGE	36	0.461	0.125	0.010^‡^
	IGG_1_	36	0.393	0.089	
Shrimp	IGE	36	0.196	0.057	0.631^‡^
	IGG_1_	36	0.203	0.055	
Egg	IGE	36	0.217	0.092	0.414^£^
	IGG_1_	36	0.191	0.060	
Soy	IGE	36	0.219	0.072	0.256^‡^
	IGG_1_	36	0.202	0.060	
Wheat	IGE	36	0.218	0.057	0.841^‡^
	IGG_1_	36	0.216	0.056	
Cashew nut	IGE	36	0.204	0.060	0.466^‡^
	IGG_1_	36	0.195	0.053	
Peanut	IGE	36	0.187	0.046	0.324^‡^
	IGG_1_	36	0.177	0.044	
Corn	IGE	36	0.214	0.072	0.477^‡^
	IGG_1_	36	0.203	0.053	
Cocoa	IGE	36	0.154	0.034	0.914^‡^
	IGG_1_	36	0.155	0.032	
Kiwi	IGE	36	0.171	0.035	0.591^‡^
	IGG_1_	36	0.176	0.045	
Papaya	IGE	36	0.173	0.042	0.814^‡^
	IGG_1_	36	0.176	0.050	
Banana	IGE	36	0.185	0.047	0.157^‡^
	IGG_1_	36	0.169	0.048	
Tomato	IGE	36	0.165	0.042	0.739^‡^
	IGG_1_	36	0.169	0.048	

‡—Student t test for independent samples

£—Mann-Whitney test. Significant p value less than or equal to 0.05.

When comparing the mean of IgE and IgG_1_ concentrations in saliva, all foods had no statistical difference (p > 0.05), except for cow’s milk and tomato, which presented a difference (p < 0.001, p < 0.003) (Table 4).

**Table 4:** Comparison of IgE concentration with IgG_1_ concentration in saliva in each food.

Food	Parameter	n	Average (O.D.)	Standard Deviation	p
Cow milk	IGE	36	0.485	0.076	< 0.001^‡^
	IGG_1_	36	0.333	0.067	
Fish	IGE	36	0.376	0.082	0.446^‡^
	IGG_1_	36	0.391	0.081	
Shrimp	IGE	36	0.182	0.042	0.106^‡^
	IGG_1_	36	0.198	0.042	
Egg	IGE	36	0.201	0.054	0.126^‡^
	IGG_1_	36	0.182	0.045	
Soy	IGE	36	0.216	0.062	0.235^‡^
	IGG_1_	36	0.201	0.045	
Wheat	IGE	36	0.224	0.065	0.847^‡^
	IGG_1_	36	0.221	0.051	
Cashew nut	IGE	36	0.200	0.059	0.764^‡^
	IGG_1_	36	0.204	0.051	
Peanut	IGE	36	0.184	0.047	0.571^‡^
	IGG_1_	36	0.178	0.047	
Corn	IGE	36	0.182	0.058	0.771^‡^
	IGG_1_	36	0.186	0.047	
Cocoa	IGE	36	0.152	0.028	0.492^‡^
	IGG_1_	36	0.165	0.105	
Kiwi	IGE	36	0.179	0.043	0.359^‡^
	IGG_1_	36	0.169	0.044	
Papaya	IGE	36	0.208	0.053	0.110^‡^
	IGG_1_	36	0.183	0.076	
Banana	IGE	36	0.192	0.058	0.930^‡^
	IGG_1_	36	0.191	0.049	
Tomato	IGE	36	0.162	0.037	0.003^£^
	IGG_1_	36	0.197	0.057	

‡—Student t test for independent samples

£—Mann-Whitney test. Significant p value less than or equal to 0.05.

When analyzing the mean of IgE concentration against shrimp, egg, soybeans, wheat, chestnut, peanut, kiwi, banana, tomato and cocoa; no significantly difference between Serum and Saliva was found (p > 0.05). However, the mean of IgE concentration against cow’s milk and papaya in serum was significantly lower than in Saliva (p < 0.05). In addition, the mean of IgE concentration against fish and corn in serum was significantly higher than in Saliva (p < 0.05) (Table 5).

**Table 5:** Comparison of IgE concentration between serum and saliva in each food.

Food	Biological compartment	n	Average (O.D.)	Standard deviation	p
Cow milk	Serum	36	0.430	0.078	0.004^‡^
	Saliva	36	0.485	0.076	
Fish	Serum	36	0.461	0.125	0.016^£^
	Saliva	36	0.376	0.082	
Shrimp	Serum	36	0.196	0.057	0.421^£^
	Saliva	36	0.182	0.042	
Egg	Serum	36	0.217	0.092	0.987^£^
	Saliva	36	0.201	0.054	
Soy	Serum	36	0.219	0.072	0.849^‡^
	Saliva	36	0.216	0.062	
Wheat	Serum	36	0.218	0.057	0.712^‡^
	Saliva	36	0.224	0.065	
Cashew nut	Serum	36	0.204	0.060	0.740^‡^
	Saliva	36	0.200	0.059	
Peanut	Serum	36	0.187	0.046	0.761^‡^
	Saliva	36	0.184	0.047	
Corn	Serum	36	0.214	0.072	0.043^‡^
	Saliva	36	0.182	0.058	
Cocoa	Serum	36	0.154	0.034	0.811^‡^
	Saliva	36	0.152	0.028	
Kiwi	Serum	36	0.171	0.035	0.377^‡^
	Saliva	36	0.179	0.043	
Papaya	Serum	36	0.173	0.042	0.006^£^
	Saliva	36	0.208	0.053	
Banana	Serum	36	0.185	0.047	0.561^‡^
	Saliva	36	0.192	0.058	
Tomato	Serum	36	0.165	0.042	0.734^‡^
	Saliva	36	0.162	0.037	

‡—Student t test for independent samples

£—Mann-Whitney test. Significant p value less than or equal to 0.05.

Table 6 shows that the mean concentration of IgG_1_ in cow’s milk, fish, shrimp, egg, soy, wheat, brown, peanut, kiwi, papaya, banana, maize and cocoa did not differ significantly between serum and saliva (p > 0,05). But the mean IgG_1_ concentration of the tomato in the Serum was significantly lower than in Saliva (p < 0,05).

**Table 6:** Comparison of IgG_1_ concentration between serum and saliva in each food.

Food	Biological compartment	n	Average (O.D.)	Standard deviation	p
Cow milk	Serum	36	0.339	0.048	0.654^‡^
	Saliva	36	0.333	0.067	
Fish	Serum	36	0.393	0.089	0.910^‡^
	Saliva	36	0.391	0.081	
Shrimp	Serum	36	0.203	0.055	0.696^‡^
	Saliva	36	0.198	0.042	
Egg	Serum	36	0.191	0.060	0.503^‡^
	Saliva	36	0.182	0.045	
Soy	Serum	36	0.202	0.060	0.975^‡^
	Saliva	36	0.201	0.045	
Wheat	Serum	36	0.216	0.056	0.675^‡^
	Saliva	36	0.221	0.051	
Cashew nut	Serum	36	0.195	0.053	0.463^‡^
	Saliva	36	0.204	0.051	
Peanut	Serum	36	0.177	0.044	0.934^‡^
	Saliva	36	0.178	0.047	
Corn	Serum	36	0.203	0.053	0.142^‡^
	Saliva	36	0.186	0.047	
Cocoa	Serum	36	0.155	0.032	0.589^‡^
	Saliva	36	0.165	0.105	
Kiwi	Serum	36	0.176	0.045	0.546^‡^
	Saliva	36	0.169	0.044	
Papaya	Serum	36	0.176	0.050	0.648^‡^
	Saliva	36	0.183	0.076	
Banana	Serum	36	0.169	0.048	0.055^‡^
	Saliva	36	0.191	0.049	
Tomato	Serum	36	0.169	0.048	0.010^£^
	Saliva	36	0.197	0.057	

‡—Student t test for independent samples

£—Mann-Whitney test. Significant p value less than or equal to 0.05.

## Discussion

The diagnosis of food allergy by the detection of specific IgE and IgG_1_ immunoglobulins using saliva was reported in this study, making this article the first work that reports the possibility of a noninvasive diagnosis for food allergy. In addition, the interaction of allergenic proteins with serum and saliva can be observed by detecting both IgE and IgG_1_.

High levels of salivary IgE and IgG_1_ specific for precipitated cow’s milk proteins from 36 allergic children were detected by ELISA using the RAST test. Likewise, 100% of the experimental group also presented high levels of IgE and IgG_1_ specific for the fish. Although fish is not usually used in the feeding of children in early childhood, the result suggests that these children have become sensitized by other routes of immunization other than oral, since fish is a food widely used by the population of Ceará. This is according to SICHERER et al. (2011) [15] which showed that most peanut allergy children experience their first allergic reaction to peanuts at the first intake, suggesting that the sensitization that resulted in IgE production must have occurred through exposure through a non-oral route. Two leading theories for the basis of such sensitization are *in utero* sensitization, or by home exposure through non-oral routes.

When analyzed with maize extract, the serum and salivary levels of specific IgE were observed in 36% and 39% of the samples, repectively. Similarly, IgG_1_ were detected in 25% in the allergic group. Corn is a cereal widely used in Brazilian food, however, few studies have addressed specific allergic reactions to this food. Nonetheless, in one of them, performed in a multicenter study in eight different regions of Brazil (n = 306), being tested by means of the *prick test*, Rizzo et al (1995) [16] found a prevalence of 10.1% positive cutaneous reactivity for corn in the northeast region.

When soybean was used as antigen, the presence of specific serum IgE and IgG_1_ were detected in 52% and 55% respectively and salivary IgE and IgG_1_ in 55% and 53% of the allergic group. Similarly, for peanuts, serum IgE and IgG_1_ reactivity were 36% and 39%, respectively; IgE and IgG_1_ salivary 25% and 25% respectively. Still in Rizzo’s study et al (1995) [16] a prevalence of 10.1% of cutaneous reactivity was also found for soybean and 8.4% for egg and peanut in northeastern Brazil.

For the egg white the presence of IgE and IgG_1_ in the serum was 39% and 60%, respectively; while in saliva, IgE was observed in 55% and IgG_1_ in 44%. When comparing specific IgE and IgG_1_ levels in serum and saliva for the foods in this study, there was no significant difference.

These results demonstrated that, when comparing the levels of specific IgE and IgG_1_ immunoglobulins in the serum and saliva for the foods tested, no statistical difference between them are found for most foods.

Although IgE is the immunoglobulin identified as the mediator of food allergic reactions, several evidences show that systemic anaphylaxis can also be induced by IgG class immunoglobulins [6]. Mast cells can respond to both IgE and IgG antibodies. IgG antibodies in FA can influence the formation of immunocomplexes contradicting the traditional concept that these antibodies inhibit allergic processes [17]. Therefore, immunoglobulin G serves as a mediating effect rather than inhibiting the hypersensitivity reaction caused by food intolerance. Due to the great diversity of allergenic food products, it is believed that immunological reactions may be very different and their mechanisms may work in a more complicated way, requiring further studies [18].

Corroborating with the theory of IgG participate in the allergic process, Finkelman, Khodoun and Strait (2016) [6], observed in their study with mice that human IgG_1_, IgG_2_ and IgG_4_ can bind to FcγRIII high affinity receptors, activating mast cells, basophils, neutrophils and macrophages, which leads to the production of PAF (platelet activation factor) in anaphylaxis in humans.

Both IgE and IgG antibodies are found in the serum of allergic patients, but the relative concentration of the various classes is poorly understood and rarely investigated. This may have important therapeutic consequences if, in fact, neutrophils can induce IgG-dependent anaphylactic reactions in humans [19]. The allergen-specific IgG_1_ detection results shown in this study are relevant, since they show the specific IgE and IgG_1_ detection for different foods studied in serum and saliva. In addition, this result shows that saliva can be used to diagnose FA.

Several researchers have been demonstrating the use of saliva for diagnosis of different diseases. As can be observed in the Luzza et al (1997) [20] study who compared salivary IgG levels to diagnose Helicobacter Pilory infections with the gold standard diagnostic test, finding significantly higher salivary IgG levels in people with HP infection when compared to the control group, presenting specificity and sensitivity of the salivary test of 82% and 93%.

According to the study by Cai et al. (2014) [21], a high prevalence of serum IgG antibodies to food allergens were found in allergic patients presenting intestinal symptoms, also reporting that this immunoglobulin is a potent indicator for the state of the allergic disease facilitating orientations during the diet. However, Mekkel et al (2005) [22] considers according to his study that IgG is present, in significant amounts, rarely in immediate type reactions, being considered as the best indicator in this type of reaction to immunoglobulin E. Complementing the previous study, circulating IgG was shown to give a more delayed or even asymptomatic response following exposure to a single food in the Crowe and Perdue study (1992) [23], which report that IgG mediated reactions have a slower response after antigen exposure.

In the study made by Hochwallner et al. (2011) [24], it was investigated whether patients with non-IgE-mediated APLV can be distinguished from non-allergic individuals through serum IgA levels and IgG subclasses, showing higher levels of IgG_1_ and IgG_4_ for some types of caseins and α-lactoglobulin.

The test to diagnose FA currently considered gold standard is the oral provocation test, but it presents risks, a high cost and there are no practical alternatives, parameters and clear guidelines to discriminate which children should take this test [25]. In contrast, Volppi and Maccari (2009) [25] showed in their study that the ELISA technique is highly sensitive and specific for detecting the presence of serum antibodies, through IgE and IgG isotope tests for 160 different dietary proteins in 6879 individuals, technique is considered low cost and does not cause allergic manifestations in the patient.

According to Hiller et al (2002) [26], the ELISA test allows to verify the profile of reactivity and the detection of allergic components by specific IgE in small amounts of serum but can not identify the allergic disease. But, according to Ramos, Lyra, Oliveira (2013) [27] the ELISA test is a less expensive, simpler and more sensitive feature used by many laboratories to test the presence of antibodies in patients allergic to numerous food antigens, since these have high levels of some IgG subtypes. In addition, some companies also produce kits for the detection of IgE, which, together with clinical examination, will aid in the selection of allergenic foods to be avoided by the patient.

Vojdani A. and Vojodani C. (2015) [28] report that some people are sensitive to lipids, and currently the skin tests are performed only with protein components dissolved in water, and may produce false negative results, making it fundamental to develop a methodology for this type of evaluation.

Therefore, Vojdani A. and Vojodani C. (2015) [28] stated that serological tests measuring both levels of IgG and IgE would make possible a diagnosis for more reliable allergy test, making it possible to diagnose both IgE-mediated, non-mediated and mixed allergies.

## Final considerations

It was possible to verify with this study the similarity in the specificity and sensitivity that the ELISA test, using both serum and saliva for the detection of IgE and IgG_1_, presented for the RAST test.

In this context, it was found that the use of saliva for the detection of IgE is more sensitive to diagnose CMPA alone because it has a higher amount, however IgG_1_ also showed efficiency in the diagnosis of food allergy through saliva.

The use of salivary combined with the method to diagnose food allergy is suggested, with IgE and IgG_1_ being measured in order to obtain more reliable results as well as to investigate mediated, non-IgE and mixed mediated allergies.

## Supporting information

S1 File Research instrument. (PDF)

S2 File Statistical Analysis Report. (PDF)

S3 File Closed free consent term. (PDF)

S4 File Raw data test diagnostic allergy. (XLSX)

S5 File Suplementary I. (DOCX)
