# Supplementary material for: Detection of serum and salivary IgE and IgG1 immunoglobulins specific for diagnosis of food allergy
Source: PLoS One. 2019 Apr 17;14(4):e0214745. doi: 10.1371/journal.pone.0214745 (PMC6469776; doi:10.1371/journal.pone.0214745)
Supplement: S1 File — (PDF) [file pone.0214745.s001.pdf]

PROTOCOLO DE INVESTIGAÇÃO DE ALERGIA ALIMENTAR

PROTOCOL FOR FOOD ALLERGY RESEARCH

NOME (NAME):____
PRONTUÁRIO (PRONOUNCE NUMBER): ___________________________

DN(date of birth**):**____
IDADE ATUAL (AGE):____

DIAGNÓSTICO PATOLÓGICO (diagnosis):__________________________

POR FAVOR RESPONDER AS PERGUNTAS ABAIXO (PLEASE ANSWER THE QUESTIONS BELOW) **:**

1- Porque procurou atendimento no Hospital Albert Sabin?
1- Why did you seek care at Albert Sabin Hospital?

____
____

2- Quais os sintomas que está apresentando?
2- What are the symptoms you are presenting?

____
____

3- Está tomando algum medicamento, ou tomou essa semana?
3-Are you taking any medication, or did you take it this week?

____
____

4- Tem alguma alergia à alimentos e/ou medicamentos?
4- Do you have any allergies to food and / or medications?

____
____

5- Tem Rinite, sinusite, faringite alérgia?
5 - Do you have rhinitis, sinusitis, allergic pharyngitis?

____
____

6- Você já comeu os alimentos abaixo, e com qual frequência?
6-Have you eaten the food below, and how often?

Milho ou derivados:____
Sim ( ) Não ( ) - Apenas 1x ( ) Mais de 3x ( ) Come sempre ( )
Apresentou alguma reação alérgica: Sim ( ) Não ( )
Se SIM qual? ( )Urticária ( )Diarreia ( )Vômitos/Náusea ( )Dermatite
Outros:____

Maize or derivatives: ____
Yes () No () - Only 1x () More than 3x () Always eats ()
Has any allergic reaction: Yes () No ()
If YES, which one? () Urticaria () Diarrhea () Vomiting / Nausea () Dermatitis
Others:____

Mamão ou derivados:_______________________________________
Sim ( ) Não ( ) - Apenas 1x ( ) Mais de 3x ( ) Come sempre ( )
Apresentou alguma reação alérgica: Sim ( ) Não ( )
Se SIM qual? ( )Urticária ( )Diarreia ( )Vômitos/Náusea ( )Dermatite
Outros:____

Papaya or derivatives: _______________________________________
Yes () No () - Only 1x () More than 3x () Always eats ()
Has any allergic reaction: Yes () No ()
If YES, which one? () Urticaria () Diarrhea () Vomiting / Nausea () Dermatitis
Others:____

Leite de vaca ou derivados:__________________________________
Sim ( ) Não ( ) - Apenas 1x ( ) Mais de 3x ( ) Come sempre ( )
Apresentou alguma reação alérgica: Sim ( ) Não ( )
Se SIM qual? ( )Urticária ( )Diarreia ( )Vômitos/Náusea ( )Dermatite
Outros:____

Cow's Milk or Derivatives: __________________________________
Yes () No () - Only 1x () More than 3x () Always eats ()
Has any allergic reaction: Yes () No ()
If YES, which one? () Urticaria () Diarrhea () Vomiting / Nausea () Dermatitis
Others:____

Ovo de galinha ou derivados:__________________________________
Sim ( ) Não ( ) - Apenas 1x ( ) Mais de 3x ( ) Come sempre ( )
Apresentou alguma reação alérgica: Sim ( ) Não ( )
Se SIM qual? ( )Urticária ( )Diarreia ( )Vômitos/Náusea ( )Dermatite
Outros:____

Chicken d egg or derivatives: __________________________________
Yes () No () - Only 1x () More than 3x () Always eats ()
Has any allergic reaction: Yes () No ()
If YES, which one? () Urticaria () Diarrhea () Vomiting / Nausea () Dermatitis
Others:____

Soja ou derivados:____
Sim ( ) Não ( ) - Apenas 1x ( ) Mais de 3x ( ) Come sempre ( )
Apresentou alguma reação alérgica: Sim ( ) Não ( )
Se SIM qual? ( )Urticária ( )Diarreia ( )Vômitos/Náusea ( )Dermatite
Outros:____

Soy or derivatives: ____
Yes () No () - Only 1x () More than 3x () Always eats ()
Has any allergic reaction: Yes () No ()
If YES, which one? () Urticaria () Diarrhea () Vomiting / Nausea () Dermatitis
Others:____

Amendoim ou derivados:_______________________________________
Sim ( ) Não ( ) - Apenas 1x ( ) Mais de 3x ( ) Come sempre ( )
Apresentou alguma reação alérgica: Sim ( ) Não ( )
Se SIM qual? ( )Urticária ( )Diarreia ( )Vômitos/Náusea ( )Dermatite
Outros:____

Peanuts or derivatives: _______________________________________
Yes () No () - Only 1x () More than 3x () Always eats ()
Has any allergic reaction: Yes () No ()
If YES, which one? () Urticaria () Diarrhea () Vomiting / Nausea () Dermatitis
Others:____

Já fez algum exame para detectar alergia? ( )Sim ( )Não
Qual?____

Have you done any tests to detect allergy? () Yes () No Which one?

ANTECEDENTES FAMILIARES (FAMILY BACKGROUND:**):**

MÃE
(Mom):____
____
PAI
(Dad):____
____
IRMÃOS
(Brothers):____
____

PROTOCOLO DE HIPERSENSIBILIDADE ALIMENTAR

PROTEOL OF FOOD HYPERSENSITIVITY

NOME (NAME):____
PRONTUÁRIO (PRONOUNCE NUMBER): _________________________

DN(date of birth**):**____
IDADE ATUAL (AGE):______________________________________

DIAGNÓSTICO PATOLÓGICO (diagnosis):____________________________
MÃE (Mom):____

PAI (Dad):____

TELEFONE (Phone):______________________________**DATA:**_____/______/______

ALEITAMENTO MATERNO (breastfeeding**): (   ) Yes, Exclusive** ____________________
(   ) MIXTA ATÉ:____________________
(   ) No

QUEIXA PRINCIPAL (main complaint**):.........................MARCAR COM UM X..........................**

DOENÇAS<br>Diseases	IDADE DO INÍCIO<br>age of onset	DESENCANDEANTES<br>suspicious food	TRATAMENTO DIETA/LEITE<br>Tratament
URTICÁRIA			
ANGIODEMA			
DERMATITE			
VÔMITOS			
DIARREIA			
DOR ABDOMINAL			
BAIXO GANHO DE PESO			
SANGRAMENTOS			
LARINGITES			
OUTROS			

COMORBIDADES (comorbidities**):**
____
____
____

OUTRAS ALERGIAS (other allergies**):**
____
____
____

ANTECEDENTES FAMILIARES (FAMILY BACKGROUND**):**

MÃE (Mom):____
PAI (Dad):____
IRMÃOS
(Brothers):____

HISTÓRIA ALIMENTAR (food history**)**

INTRODUÇÃO DE SÓLIDOS (introduction o solids**):**_________________________________

DIETA ATUAL (current diet and meals**):**

CAFÉ:____
LANCHE:____
ALMOÇO:____
LANCHE:____
JANTAR:____
NOITE:____
OUTROS:____

EXAME FÍSICO

PESO:___________________________**PERCENTIL:**_______________________
ESTATURA:______________________**PERCENTIL:**_______________________
TURGOR:____
PREGAS:____
ABDOME:____

PHYSICAL EXAM

WEIGHT: ___________________________ PERCENTILE: _______________________
STATUS: ___________________________ PERCENTILE: _______________________
TURGOR: ____
PREGAS: ____
ABDOME: ____

AVALIAÇÃO LABORATORIAL (LABORATORY EVALUATION:**):**

IgE:
IgA:
Ac Anti-transglutaminase:
Ac Anti-gliadina:
Ac Anti-endomisio:
Rast para alimentos ou PICK:
Alfa-1-antitripsina:
Bx com contagem de eosinófilos:
Bx com contagem de eosinófilos:
EDA:
COLONOSCOPIA:
PHMETRIA:

CONDUTA:____
____
RETORNO:____

CONDUCT:____
____
RETURN: ____
