# Supplementary material for: Detection of serum and salivary IgE and IgG1 immunoglobulins specific for diagnosis of food allergy
Source: PLoS One. 2019 Apr 17;14(4):e0214745. doi: 10.1371/journal.pone.0214745 (PMC6469776; doi:10.1371/journal.pone.0214745)
Supplement: S2 File — (PDF) [file pone.0214745.s002.pdf]

RELATÓRIO DE ANÁLISE ESTATÍSTICA
STATISTICAL ANALYSIS REPORT

1-METODOLOGIA ESTATÍSTICA
1-STATISTICAL METHODOLOGY

As análises estatísticas deste trabalho foram realizadas no software R versão 3.4.1. Os dados foram expressos como medidas de tendência central e dispersão. Também foram apresentados gráficos de barra de erros com a média e o intervalo de confiança de 95%. Testou-se a normalidade das variáveis por meio do teste de Kolmogorov-Smirnov e homogeneidade das variâncias pelo teste de Levene. Para a comparação de médias utilizou-se o teste t de Student para amostras independentes, quando os dados foram normais e homogêneos, e o teste de Mann-Whitney, quando os dados foram não normais e não homogêneos. Para a comparação entre mais de duas médias, quando os dados apresentaram-se normais e homogêneos utilizou-se o teste de ANOVA, e quando não, o teste de Kruskal-Wallis, os quais foram seguidos pelo pós-teste de Tukey. Para a comparação dos valores médios das variáveis com o valor de referência utilizou-se o teste t de Student para uma amostra. Os resultados foram considerados significativos com valores de p abaixo de 0,05.

The statistical analyzes of this work were carried out in software R version 3.4.1. Data were expressed as measures of central tendency and dispersion. Also, error bar graphs with mean and 95% confidence intervals were presented. Normality of variables was tested using the Kolmogorov-Smirnov test and homogeneity of variances by the Levene test. For the comparison of means, the Student's t-test was used for independent samples, when the data were normal and homogeneous, and the Mann-Whitney test, when the data were non-normal and non-homogeneous. For the comparison of more than two means, when the data were normal and homogeneous, we used the ANOVA test and, if not, the Kruskal-Wallis test, which were followed by the Tukey post-test. For the comparison of the mean values of the variables with the reference value, Student's t-test was used for one sample. The results were considered significant with p values below 0.05.

2 – RESULTADOS

2 - *RESULTS*

Quadro 1. Comparação da concentração de IGE entre o soro e a saliva em cada alimento

Table 1. Comparison of the GGE concentration between serum and saliva in each feed

Alimento	Compartimento biológico	n	Média	Desvio Padrão	p
Leite de vaca	Soro	36	0,430	0,078	0,004‡
	Saliva	36	0,485	0,076	
Peixe	Soro	36	0,461	0,125	0,016£
	Saliva	36	0,376	0,082	
Camarão	Soro	36	0,196	0,057	0,421£
	Saliva	36	0,182	0,042	
Ovo	Soro	36	0,217	0,092	0,987£
	Saliva	36	0,201	0,054	
Soja	Soro	36	0,219	0,072	0,849‡
	Saliva	36	0,216	0,062	
Trigo	Soro	36	0,218	0,057	0,712‡
	Saliva	36	0,224	0,065	
Castanha	Soro	36	0,204	0,060	0,740‡
	Saliva	36	0,200	0,059	
Amendoim	Soro	36	0,187	0,046	0,761‡
	Saliva	36	0,184	0,047	
Kiwi	Soro	36	0,171	0,035	0,377‡
	Saliva	36	0,179	0,043	
Mamão	Soro	36	0,173	0,042	0,006£
	Saliva	36	0,208	0,053	
Banana	Soro	36	0,185	0,047	0,561‡
	Saliva	36	0,192	0,058	
Tomate	Soro	36	0,165	0,042	0,734‡
	Saliva	36	0,162	0,037	
Milho	Soro	36	0,214	0,072	0,043‡
	Saliva	36	0,182	0,058	
Cacau	Soro	36	0,154	0,034	0,811‡
	Saliva	36	0,152	0,028	

Legenda: ‡ - teste t de Student para amostras independentes; £ - teste te Mann-Whitney. Valor de p considerado significativo menor ou igual a 0,05.

Comentário do quadro 1:

- A concentração média de IGE do camarão, do ovo, da soja, do trigo, da castanha, do amendoim, do kiwi, da banana, do tomate e do cacau não difeiru significativamente entre o Soro e a Saliva (p > 0,05).

- A concentração média de IGE do leite de vaca e do mamão no Soro foi significativamente menor que na Saliva (p < 0,05).
- A concentração média de IGE do peixe e do milho no Soro foi significativamente maior que na Saliva (p < 0,05).

Comment of table 1:

- The mean GGE concentration of shrimp, egg, soybean, wheat, peanut, peanut, kiwi, banana, tomato and cocoa did not differ significantly between serum and saliva (p> 0.05).
- The mean GGE concentration of cow's milk and papaya in serum was significantly lower than in Saliva (p <0.05).
- The mean GGE concentration of fish and corn in serum was significantly higher than in saliva (p <0.05).

Quadro 2. Comparação da concentração de IGG1 entre o soro e a saliva em cada alimento

Table 2. Comparison of IGG1 concentration between serum and saliva in each feed

Alimento	Compartimento biológico	n	Média	Desvio Padrão	p
Leite de vaca	Soro	36	0,339	0,048	0,654‡
	Saliva	36	0,333	0,067	
Peixe	Soro	36	0,393	0,089	0,910‡
	Saliva	36	0,391	0,081	
Camarão	Soro	36	0,203	0,055	0,696‡
	Saliva	36	0,198	0,042	
Ovo	Soro	36	0,191	0,060	0,503‡
	Saliva	36	0,182	0,045	
Soja	Soro	36	0,202	0,060	0,975‡
	Saliva	36	0,201	0,045	
Trigo	Soro	36	0,216	0,056	0,675‡
	Saliva	36	0,221	0,051	
Castanha	Soro	36	0,195	0,053	0,463‡
	Saliva	36	0,204	0,051	
Amendoim	Soro	36	0,177	0,044	0,934‡
	Saliva	36	0,178	0,047	
Kiwi	Soro	36	0,176	0,045	0,546‡
	Saliva	36	0,169	0,044	

Mamão	Soro	36	0,176	0,050	0,648‡
	Saliva	36	0,183	0,076	
Banana	Soro	36	0,169	0,048	0,055‡
	Saliva	36	0,191	0,049	
Tomate	Soro	36	0,169	0,048	0,010£
	Saliva	36	0,197	0,057	
Milho	Soro	36	0,203	0,053	0,142‡
	Saliva	36	0,186	0,047	
Cacau	Soro	36	0,155	0,032	0,589‡
	Saliva	36	0,165	0,105	

Legenda: ‡ - teste t de Student para amostras independentes; £ - teste te Mann-Whitney.
Valor de p considerado significativo menor ou igual a 0,05.

Comentário do quadro 2:
- A concentração média de IGG1 do leite de vaca, do peixe, do camarão, do ovo, da soja, do trigo, da castanha, do amendoim, do kiwi, do mamão, da banana, do milho e do cacau não difeiru significativamente entre o Soro e a Saliva (p > 0,05).
- A concentração média de IGG1 do tomate no Soro foi significativamente menor que na Saliva (p < 0,05).

Comment of table 2:
- The average IGG1 concentration of cow's milk, fish, shrimp, egg, soybeans, wheat, chestnut, peanut, kiwi, papaya, banana, maize and cocoa did not differ significantly between Serum and Saliva (p> 0.05).
- The mean serum IGG1 concentration in the serum was significantly lower than in Saliva (p <0.05).

Quadro 3. Comparação da concentração de IGE com a concentração de IGG1 no soro em cada alimento

Table 3. Comparison of GGE concentration with serum IGG1 concentration in each feed

Alimento	Parâmetro	n	Média	Desvio Padrão	p
Leite de vaca	IGE	36	0,430	0,078	< 0,001£
	IGG1	36	0,339	0,048	
Peixe	IGE	36	0,461	0,125	0,010‡
	IGG1	36	0,393	0,089	
Camarão	IGE	36	0,196	0,057	0,631‡
	IGG1	36	0,203	0,055	

Ovo	IGE	36	0,217	0,092	0,414£
	IGG1	36	0,191	0,060	
Soja	IGE	36	0,219	0,072	0,256‡
	IGG1	36	0,202	0,060	
Trigo	IGE	36	0,218	0,057	0,841‡
	IGG1	36	0,216	0,056	
Castanha	IGE	36	0,204	0,060	0,466‡
	IGG1	36	0,195	0,053	
Amendoim	IGE	36	0,187	0,046	0,324‡
	IGG1	36	0,177	0,044	
Kiwi	IGE	36	0,171	0,035	0,591‡
	IGG1	36	0,176	0,045	
Mamão	IGE	36	0,173	0,042	0,814‡
	IGG1	36	0,176	0,050	
Banana	IGE	36	0,185	0,047	0,157‡
	IGG1	36	0,169	0,048	
Tomate	IGE	36	0,165	0,042	0,739‡
	IGG1	36	0,169	0,048	
Milho	IGE	36	0,214	0,072	0,477‡
	IGG1	36	0,203	0,053	
Cacau	IGE	36	0,154	0,034	0,914‡
	IGG1	36	0,155	0,032	

Legenda: ‡ - teste t de Student para amostras independentes; £ - teste te Mann-Whitney. Valor de p considerado significativo menor ou igual a 0,05.

Comentário do quadro 3:

- No soro, a concentração de IGE e IGG1 do camarão, do ovo, da soja, do trigo, da castanha, do amendoim, do kiwi, do mamão, da banana, do tomate, do milho e do cacau não diferiu significativamente (p > 0,05).
- No soro, a concentração de IGE do leite de vaca e do peixe foi significativamente maior que a concentração de IGG1 (p < 0,05).

Comment of table 3:

- In the serum, the concentration of GI and IGG1 of shrimp, egg, soybean, wheat, chestnut, peanut, kiwi, papaya, banana, tomato, maize and cocoa did not differ significantly (p > 0.05).
- In serum, the concentration of GGE in cow's milk and fish was significantly higher than the concentration of IGG1 (p <0.05).

Quadro 4. Comparação da concentração de IGE com a concentração de IGG1 na saliva em cada alimento

Table 4. Comparison of GGE concentration with IGG1 concentration in saliva in each feed

Alimento	Parâmetro	n	Média	Desvio Padrão	p
Leite de vaca	IGE	36	0,485	0,076	< 0,001‡
	IGG1	36	0,333	0,067	
Peixe	IGE	36	0,376	0,082	0,446‡
	IGG1	36	0,391	0,081	
Camarão	IGE	36	0,182	0,042	0,106‡
	IGG1	36	0,198	0,042	
Ovo	IGE	36	0,201	0,054	0,126‡
	IGG1	36	0,182	0,045	
Soja	IGE	36	0,216	0,062	0,235‡
	IGG1	36	0,201	0,045	
Trigo	IGE	36	0,224	0,065	0,847‡
	IGG1	36	0,221	0,051	
Castanha	IGE	36	0,200	0,059	0,764‡
	IGG1	36	0,204	0,051	
Amendoim	IGE	36	0,184	0,047	0,571‡
	IGG1	36	0,178	0,047	
Kiwi	IGE	36	0,179	0,043	0,359‡
	IGG1	36	0,169	0,044	
Mamão	IGE	36	0,208	0,053	0,110‡
	IGG1	36	0,183	0,076	
Banana	IGE	36	0,192	0,058	0,930‡
	IGG1	36	0,191	0,049	
Tomate	IGE	36	0,162	0,037	0,003£
	IGG1	36	0,197	0,057	
Milho	IGE	36	0,182	0,058	0,771‡
	IGG1	36	0,186	0,047	
Cacau	IGE	36	0,152	0,028	0,492‡
	IGG1	36	0,165	0,105	

Legenda: ‡ - teste t de Student para amostras independentes; £ - teste te Mann-Whitney. Valor de p considerado significativo menor ou igual a 0,05.

Comentário do quadro 4:

- Na saliva, a concentração de IGE e IGG1 do leite de vaca, do peixe, do camarão, do ovo, da soja, do trigo, da castanha, do amendoim, do kiwi, do mamão, da banana, do milho e do cacau não diferiu significativamente (p > 0,05).

- Na saliva, a concentração de IGE do tomate foi significativamente menor que a concentração de IGG1 ($p < 0,05$).

Comment of table 4:

- In saliva, the concentration of GI and IGG1 in cows' milk, fish, shrimp, egg, soybeans, wheat, chestnut, peanut, kiwi, papaya, banana, maize and cocoa did not differ significantly ($p > 0.05$).
- In the saliva, the GGE concentration of the tomato was significantly lower than the IGG1 concentration ($p < 0.05$).

Quadro 5. Comparação das variáveis com o cuttoff em cada alimento

Table 5. Comparison of the variables with the cuttoff in each food

Alimento	Parâmetro	n	Mínimo	Máximo	Média	Desvio Padrão	Cuttoff	p
Leite de vaca	IGE no Soro	36	0,249	0,638	0,430	0,078	0,135	< 0,001
	IGE na Saliva	36	0,363	0,752	0,485	0,076	0,137	< 0,001
	IGG1 no soro	36	0,266	0,554	0,339	0,048	0,140	< 0,001
	IGG1 na saliva	36	0,235	0,566	0,333	0,067	0,142	< 0,001
Peixe	IGE no Soro	36	0,247	0,730	0,461	0,125	0,140	< 0,001
	IGE na Saliva	36	0,222	0,482	0,376	0,082	0,150	< 0,001
	IGG1 no soro	36	0,238	0,524	0,393	0,089	0,150	< 0,001
	IGG1 na saliva	36	0,211	0,508	0,391	0,081	0,191	< 0,001
Camarão	IGE no Soro	36	0,113	0,332	0,196	0,057	0,178	< 0,001
	IGE na Saliva	36	0,115	0,325	0,182	0,042	0,160	0,003
	IGG1 no soro	36	0,116	0,341	0,203	0,055	0,174	0,003
	IGG1 na saliva	36	0,128	0,301	0,198	0,042	0,164	< 0,001
Ovo	IGE no Soro	36	0,120	0,552	0,217	0,092	0,208	0,563
	IGE na Saliva	36	0,121	0,325	0,201	0,054	0,187	0,138
	IGG1 no soro	36	0,107	0,409	0,191	0,060	0,181	0,328
	IGG1 na saliva	36	0,123	0,333	0,182	0,045	0,177	0,473
Soja	IGE no Soro	36	0,120	0,411	0,219	0,072	0,213	0,593
	IGE na Saliva	36	0,131	0,358	0,216	0,062	0,212	0,669
	IGG1 no soro	36	0,112	0,411	0,202	0,060	0,192	0,348
	IGG1 na saliva	36	0,131	0,305	0,201	0,045	0,213	0,125
Trigo	IGE no Soro	36	0,124	0,358	0,218	0,057	0,215	0,731
	IGE na Saliva	36	0,123	0,411	0,224	0,065	0,212	0,287
	IGG1 no soro	36	0,120	0,358	0,216	0,056	0,218	0,801
	IGG1 na saliva	36	0,123	0,358	0,221	0,051	0,219	0,819
Castanha	IGE no Soro	36	0,122	0,384	0,204	0,060	0,226	0,038
	IGE na Saliva	36	0,122	0,358	0,200	0,059	0,223	0,024

	IGG1 no soro	36	0,123	0,355	0,195	0,053	0,223	0,003
	IGG1 na saliva	36	0,131	0,276	0,204	0,051	0,216	0,152
Amendoim	IGE no Soro	36	0,115	0,334	0,187	0,046	0,192	0,526
	IGE na Saliva	36	0,123	0,308	0,184	0,047	0,187	0,682
	IGG1 no soro	36	0,122	0,270	0,177	0,044	0,199	0,004
	IGG1 na saliva	36	0,118	0,269	0,178	0,047	0,185	0,342
Kiwi	IGE no Soro	36	0,113	0,251	0,171	0,035	0,213	< 0,001
	IGE na Saliva	36	0,112	0,266	0,179	0,043	0,220	< 0,001
	IGG1 no soro	36	0,128	0,334	0,176	0,045	0,199	0,004
	IGG1 na saliva	36	0,125	0,283	0,169	0,044	0,228	< 0,001
Mamão	IGE no Soro	36	0,123	0,267	0,173	0,042	0,188	0,042
	IGE na Saliva	36	0,126	0,288	0,208	0,053	0,193	0,098
	IGG1 no soro	36	0,123	0,356	0,176	0,050	0,197	0,016
	IGG1 na saliva	36	0,129	0,578	0,183	0,076	0,201	0,164
Banana	IGE no Soro	36	0,134	0,318	0,185	0,047	0,187	0,763
	IGE na Saliva	36	0,129	0,358	0,192	0,058	0,191	0,929
	IGG1 no soro	36	0,115	0,271	0,169	0,048	0,190	0,011
	IGG1 na saliva	36	0,120	0,294	0,191	0,049	0,187	0,646
Tomate	IGE no Soro	36	0,103	0,272	0,165	0,042	0,201	< 0,001
	IGE na Saliva	36	0,122	0,271	0,162	0,037	0,193	< 0,001
	IGG1 no soro	36	0,115	0,271	0,169	0,048	0,197	0,001
	IGG1 na saliva	36	0,108	0,288	0,197	0,057	0,185	0,198
Milho	IGE no Soro	36	0,127	0,448	0,214	0,072	0,248	0,007
	IGE na Saliva	36	0,106	0,332	0,182	0,058	0,238	< 0,001
	IGG1 no soro	36	0,133	0,289	0,203	0,053	0,243	< 0,001
	IGG1 na saliva	36	0,120	0,296	0,186	0,047	0,231	< 0,001
Cacau	IGE no Soro	36	0,108	0,246	0,154	0,034	0,194	< 0,001
	IGE na Saliva	36	0,104	0,260	0,152	0,028	0,200	< 0,001
	IGG1 no soro	36	0,107	0,288	0,155	0,032	0,191	< 0,001
	IGG1 na saliva	36	0,108	0,757	0,165	0,105	0,199	0,057

Legenda: ‡ - teste t de Student para uma amostra. Valor de p considerado significativo menor ou igual a 0,05.

Comentário do quadro 5:

- A concentração média de IGE no Soro, de IGE na Saliva, de IGG1 no Soro e de IGG1 na Saliva do leite de vaca foi significativamente maior que o Cuttoff desses parâmetros (p < 0,05).
- A concentração média de IGE no Soro, de IGE na Saliva, de IGG1 no Soro e de IGG1 na Saliva do peixe foi significativamente maior que o Cuttoff desses parâmetros (p < 0,05).
- A concentração média de IGE no Soro, de IGE na Saliva, de IGG1 no Soro e de IGG1 na Saliva do camarão foi significativamente maior que o Cuttoff desses parâmetros (p < 0,05).

- A concentração média de IGE no Soro, de IGE na Saliva, de IGG1 no Soro e de IGG1 na Saliva do ovo não diferiu significativamente do Cuttoff desses parâmetros ($p > 0,05$).
- A concentração média de IGE no Soro, de IGE na Saliva, de IGG1 no Soro e de IGG1 na Saliva da soja não diferiu significativamente do Cuttoff desses parâmetros ($p > 0,05$).
- A concentração média de IGE no Soro, de IGE na Saliva, de IGG1 no Soro e de IGG1 na Saliva do trigo não diferiu significativamente do Cuttoff desses parâmetros ($p > 0,05$).
- A concentração média de IGE no Soro, de IGE na Saliva e de IGG1 no Soro da castanha foi significativamente menor que o Cuttoff desses parâmetros ($p < 0,05$). A concentração média de IGG1 na Saliva não diferiu significativamente do Cuttoff desse parâmetro ($p > 0,05$).
- A concentração média de IGE no Soro, de IGE na Saliva e de IGG1 na Saliva do amendoim não diferiu significativamente do Cuttoff desses parâmetros ($p > 0,05$). A concentração média de IGG1 no Soro foi significativamente menor que o Cuttoff desse parâmetro ($p < 0,05$).
- A concentração média de IGE no Soro, de IGE na Saliva, de IGG1 no Soro e de IGG1 na Saliva do kiwi foi significativamente menor que o Cuttoff desses parâmetros ($p < 0,05$).
- A concentração média de IGE na Saliva e de IGG1 na Saliva do mamão não diferiu significativamente do Cuttoff desses parâmetros ($p > 0,05$). A concentração média de IGE no Soro e IGG1 no Soro foi significativamente menor que o Cuttoff desse parâmetro ($p < 0,05$).
- A concentração média de IGE no Soro, de IGE na Saliva e de IGG1 na Saliva da banana não diferiu significativamente do Cuttoff desses parâmetros ($p > 0,05$). A concentração média de IGG1 no Soro foi significativamente menor que o Cuttoff desse parâmetro ($p < 0,05$).
- A concentração média de IGE no Soro, de IGE na Saliva e de IGG1 no Soro do tomate foi significativamente menor que o Cuttoff desse parâmetro ($p < 0,05$). A concentração média de IGG1 na Saliva não diferiu significativamente do Cuttoff desses parâmetros ($p > 0,05$).

- A concentração média de IGE no Soro, de IGE na Saliva, de IGG1 no Soro e de IGG1 na Saliva do milho foi significativamente menor que o Cuttoff desses parâmetros ($p < 0,05$).
- A concentração média de IGE no Soro, de IGE na Saliva e de IGG1 no Soro do cacau foi significativamente menor que o Cuttoff desses parâmetros ($p < 0,05$). A concentração média de IGG1 na Saliva não diferiu significativamente do Cuttoff desse parâmetro ($p > 0,05$).

Comment of table 5:

- The mean concentration of serum GGE, GGE in Saliva, IGG1 in serum and IGG1 in cow's milk saliva was significantly higher than the cuttoff of these parameters ($p <0.05$).
- The mean concentration of serum GGE, GGE in Saliva, IGG1 in serum and IGG1 in fish saliva was significantly higher than the cuttoff of these parameters ($p <0.05$).
- The mean concentration of serum GGE, GGE in Saliva, IGG1 in serum and IGG1 in shrimp saliva was significantly higher than the cuttoff of these parameters ($p <0.05$).
- The mean concentration of serum GGE, GGE in Saliva, IGG1 in serum and IGG1 in egg saliva did not differ significantly from Cuttoff of these parameters ($p> 0.05$).
- The mean concentration of serum GGE, GGE in Saliva, IGG1 in serum and IGG1 in soybean saliva did not differ significantly from Cuttoff of these parameters ($p> 0.05$).
- The mean concentration of serum GGE, GGE in Saliva, IGG1 in serum and IGG1 in wheat saliva did not differ significantly from Cuttoff of these parameters ($p> 0.05$).
- The mean concentration of serum GGE, GGE in Saliva and IGG1 in Chestnut serum was significantly lower than the Cuttoff of these parameters ($p <0.05$). The mean concentration of IGG1 in Saliva did not differ significantly from the Cuttoff of this parameter ($p> 0.05$).
- The mean concentration of serum GGE, GGE in Saliva and IGG1 in peanut saliva did not differ significantly from Cuttoff of these parameters ($p> 0.05$). The

mean concentration of IGG1 in serum was significantly lower than the cuttoff of this parameter (p <0.05).

- The mean concentration of serum GGE, GGE in Saliva, IGG1 in serum and IGG1 in kiwi Saliva was significantly lower than the Cuttoff of these parameters (p <0.05).

The mean GGE concentration in Saliva and IGG1 in papaya Saliva did not differ significantly from Cuttoff of these parameters (p> 0.05). The mean concentration of serum GGE and IGG1 in serum was significantly lower than the cuttoff of this parameter (p <0.05).

- The mean concentration of serum GGE, GGE in Saliva and IGG1 in banana Saliva did not differ significantly from Cuttoff of these parameters (p> 0.05). The mean concentration of IGG1 in serum was significantly lower than the cuttoff of this parameter (p <0.05).
- The mean concentration of serum GGE, GGE in Saliva and IGG1 in tomato serum was significantly lower than the Cuttoff of this parameter (p <0.05). The mean concentration of IGG1 in Saliva did not differ significantly from Cuttoff of these parameters (p> 0.05).
- The mean concentration of serum GGE, GGE in Saliva, IGG1 in serum and IGG1 in corn saliva was significantly lower than the cuttoff of these parameters (p <0.05).
- The mean concentration of serum GGE, GGE in Saliva and IGG1 in Cocoa Serum was significantly lower than the Cuttoff of these parameters (p <0.05). The mean concentration of IGG1 in Saliva did not differ significantly from the Cuttoff of this parameter (p> 0.05).

Quadro 6. Comparação da concentração de IGE no Soro entre os alimentos

Table 6. Comparison of serum GGE concentration between foods

Parâmetro	Alimento	n	Média	Intervalo de confiança (95%)		p[£]
				Limite inferior	Limite superior	
IGE no Soro	Leite de vaca	36	0,430 (0,078)[a]	0,404	0,457	< 0,001
	Peixe	36	0,461 (0,125)[a]	0,419	0,503	
	Camarão	36	0,196 (0,057)[b,c,d]	0,177	0,216	
	Ovo	36	0,217 (0,092)[b,j]	0,186	0,248	
	Soja	36	0,219 (0,072)[b]	0,195	0,244	
	Trigo	36	0,218 (0,057)[b]	0,199	0,238	

Castanha	36	0,204 (0,060)[b,e]	0,184	0,225
Amendoim	36	0,187 (0,046)[b,f]	0,172	0,203
Kiwi	36	0,171 (0,035)[b,g]	0,159	0,183
Mamão	36	0,173 (0,042)[b,h]	0,159	0,187
Banana	36	0,185 (0,047)[b,i]	0,169	0,201
Tomate	36	0,165 (0,042)[d,e,f,g,h,i,j]	0,151	0,179
Milho	36	0,214 (0,072)[b,j]	0,190	0,238
Cacau	36	0,154 (0,034)[c,e,f,g,h,i]	0,142	0,165

Legenda: £ - teste de Kruskal-Wallis. Letras iguais indicam que não há diferença significativa e letras diferentes indicam que há diferença significativa entre os grupos segundo o teste de Tukey. Valor de p considerado significativo menor ou igual a 0,05.

Comentário do quadro 6:

- A concentração média de IGE do leite de vaca no Soro não diferiu significativamente da IGE do peixe no Soro (p = 0,784), mas foi significativamente maior que a concentração média de IGE de todos os outros alimentos pesquisados no Soro (p < 0,05).
- A concentração média de IGE do peixe no Soro não diferiu significativamente da IGE do leite de vaca no Soro (p = 0,784), mas foi significativamente maior que a concentração média de IGE de todos os outros alimentos pesquisados no Soro (p < 0,05).
- A concentração média de IGE do camarão no Soro foi significativamente menor que a IGE do leite de vaca e peixe no Soro (p < 0,05), mas não diferiu significativamente da concentração média de IGE dos outros alimentos pesquisados no Soro (p > 0,05).
- A concentração média de IGE do ovo no Soro foi significativamente menor que a IGE do leite de vaca e peixe no Soro e foi significativamente maior que no cacau (p < 0,05). Porém, não diferiu significativamente da IGE dos outros alimentos pesquisados no Soro (p > 0,05).
- A concentração média de IGE da soja no Soro foi significativamente menor que a IGE do leite de vaca e peixe no Soro e foi significativamente maior que no tomate e no cacau (p < 0,05). Porém, não diferiu significativamente da IGE dos outros alimentos pesquisados no Soro (p > 0,05).
- A concentração média de IGE do trigo no Soro foi significativamente menor que a IGE do leite de vaca e peixe no Soro e foi significativamente maior que no tomate e no cacau (p < 0,05). Porém, não diferiu significativamente da IGE dos outros alimentos pesquisados no Soro (p > 0,05).

- A concentração média de IGE da castanha no Soro foi significativamente menor que a IGE do leite de vaca e peixe no Soro ($p < 0,05$), mas não diferiu significativamente da concentração média de IGE dos outros alimentos pesquisados no Soro ($p > 0,05$).
- A concentração média de IGE do amendoim no Soro foi significativamente menor que a IGE do leite de vaca e peixe no Soro ($p < 0,05$), mas não diferiu significativamente da concentração média de IGE dos outros alimentos pesquisados no Soro ($p > 0,05$).
- A concentração média de IGE do kiwi no Soro foi significativamente menor que a IGE do leite de vaca e peixe no Soro ($p < 0,05$), mas não diferiu significativamente da IGE dos outros alimentos pesquisados no Soro ($p > 0,05$).
- A concentração média de IGE do mamão no Soro foi significativamente menor que a IGE do leite de vaca e peixe no Soro ($p < 0,05$), mas não diferiu significativamente da concentração média de IGE dos outros alimentos pesquisados no Soro ($p > 0,05$).
- A concentração média de IGE da banana no Soro foi significativamente menor que a IGE do leite de vaca e peixe no Soro ($p < 0,05$), mas não diferiu significativamente da IGE dos outros alimentos pesquisados no Soro ($p > 0,05$).
- A concentração média de IGE do tomate no Soro foi significativamente menor que a concentração média de IGE do leite de vaca, peixe, soja e trigo no Soro ($p < 0,05$), mas não diferiu significativamente da concentração média de IGE dos outros alimentos pesquisados no Soro ($p > 0,05$).
- A concentração média de IGE do milho no Soro foi significativamente menor que a concentração média de IGE do leite de vaca e peixe no Soro e foi significativamente maior que a concentração média de IGE do cacau no Soro ($p < 0,05$). Porém, não diferiu significativamente da concentração média de IGE dos outros alimentos pesquisados no Soro ($p > 0,05$).
- A concentração média de IGE do cacau no Soro foi significativamente menor que a concentração média de IGE do leite de vaca, peixe, ovo, soja, trigo e milho no Soro ($p < 0,05$), mas não diferiu significativamente da concentração média de IGE dos outros alimentos pesquisados no Soro ($p > 0,05$).

Comment of table 6:

- The mean serum concentration of cow's milk in the serum did not differ significantly from the GGE of the fish in the serum ($p = 0.784$), but was significantly higher than the mean GGE concentration of all other foods in the serum (p <0, 05).
- The mean serum GGE concentration of the fish did not differ significantly from the GSE of the cow's milk in the serum ($p = 0.784$), but it was significantly higher than the mean GGE concentration of all other foods investigated in the serum (p <0, 05).
- The mean serum concentration of shrimp in the serum was significantly lower than the GGE of cow's milk and fish in the serum ($p <0.05$), but did not differ significantly from the mean GGE concentration of the other foods in Soro ($p> 0.05$).
- The mean serum EGF concentration in the serum was significantly lower than the GGE of the cow's milk and fish in the serum and was significantly higher than in the cocoa ($p <0.05$). However, it did not differ significantly from the GGE of the other foods investigated in the Serum ($p> 0.05$).
- The mean serum concentration of soybean in the serum was significantly lower than the GGE of cow's milk and fish in the serum and was significantly higher than in tomato and cocoa ($p <0.05$). However, it did not differ significantly from the GGE of the other foods investigated in the Serum ($p> 0.05$).
- The mean serum GGE concentration in Whey was significantly lower than the GGE of cow's milk and fish in the Serum and was significantly higher than in tomato and cocoa ($p <0.05$). However, it did not differ significantly from the GGE of the other foods investigated in the Serum ($p> 0.05$).
- Mean serum GGE concentration in the serum was significantly lower than the GGE of cow's milk and fish in the serum ($p <0.05$), but did not differ significantly from the mean GGE concentration of the other foods tested in the serum ($p> 0.05$).
- The mean concentration of whey peanut in the serum was significantly lower than the GGE of cow's milk and fish in the serum ($p <0.05$), but did not differ significantly from the mean GGE concentration of the other foods in Soro ($p> 0.05$).

- The mean serum concentration of kiwi in the serum was significantly lower than the GGE of cow's milk and fish in the serum ($p < 0.05$), but did not differ significantly from the GGE of the other foods tested in the serum ($p > 0.05$).
- Mean serum GI concentration in the serum was significantly lower than the GGE of cow's milk and fish in the serum ($p < 0.05$), but did not differ significantly from the mean GGE concentration of the other foods tested in the serum ($p > 0.05$).
- Mean serum GI concentration of the banana in the serum was significantly lower than the GGE of cow's milk and fish in the serum ($p < 0.05$), but did not differ significantly from the GGE of the other foods tested in the serum ($p > 0.05$).
- The mean concentration of GGE of the tomato in the Serum was significantly lower than the average concentration of GSE of the cow's milk, fish, soy and wheat in the Serum ($p < 0.05$), but did not differ significantly from the average GGE concentration of the others serum levels ($p > 0.05$).
- The mean serum GGE concentration in the serum was significantly lower than the mean GGE concentration of cow's milk and fish in the serum and was significantly higher than the mean serum GI concentration in the serum ($p < 0.05$). However, it did not differ significantly from the mean GGE concentration of the other foods tested in the Serum ($p > 0.05$).
- Mean serum CGE concentration in Whey was significantly lower than the mean GGE concentration of Wheat, Fish, Egg, Soy, Wheat and Corn milk in the serum ($p < 0.05$), but did not differ significantly from the mean concentration of the other foods investigated in serum ($p > 0.05$).

Quadro 7. Comparação da concentração de IGE na Saliva entre os alimentos

Table 7. Comparison of Saliva GSE concentration between foods

Parâmetro	Alimento	n	Média	Intervalo de confiança (95%)		p£
				Limite inferior	Limite superior	
IGE na Saliva	Leite de vaca	36	0,485 (0,076)[a]	0,459	0,510	< 0,001
	Peixe	36	0,376 (0,082)[b]	0,349	0,404	
	Camarão	36	0,182 (0,042)[c,d,g]	0,168	0,196	
	Ovo	36	0,201 (0,054)[c,g,j]	0,182	0,219	
	Soja	36	0,216 (0,062)[c,g]	0,196	0,237	
	Trigo	36	0,224 (0,065)[g]	0,202	0,245	
	Castanha	36	0,200 (0,059)[c,e,g]	0,180	0,220	
	Amendoim	36	0,184 (0,047)[c,f,g]	0,168	0,200	
	Kiwi	36	0,179 (0,043)[c,h]	0,164	0,194	
	Mamão	36	0,208 (0,053)[c,g]	0,190	0,226	
	Banana	36	0,192 (0,058)[c,g,i]	0,172	0,211	
	Tomate	36	0,162 (0,037)[d,e,f,h,i,j]	0,149	0,174	
	Milho	36	0,182 (0,058)[c,g,j,k]	0,163	0,202	
	Cacau	36	0,152 (0,028)[d,f,i,k]	0,143	0,161	

Legenda: £ - teste de Kruskal-Wallis. Letras iguais indicam que não há diferença significativa e letras diferentes indicam que há diferença significativa entre os grupos segundo o teste de Tukey. Valor de p considerado significativo menor ou igual a 0,05.

Comentário do quadro 7:

- A concentração média de IGE do leite de vaca na Saliva é significativamente maior que a concentração média de IGE de todos os outros alimentos pesquisados na Saliva (p < 0,05).
- A concentração média de IGE do peixe na Saliva é significativamente menor que a concentração média de IGE do leite de vaca na Saliva (p < 0,05), mas é significativamente maior que a concentração média de IGE de todos os outros alimentos pesquisados na Saliva (p < 0,05).
- A concentração média de IGE do camarão na Saliva é significativamente menor que a concentração média de IGE do leite de vaca e peixe na Saliva (p < 0,05), mas não difere significativamente da concentração média de IGE dos outros alimentos pesquisados na Saliva (p > 0,05).
- A concentração média de IGE do ovo na Saliva é significativamente menor que a concentração média de IGE do leite de vaca e peixe na Saliva e maior que a concentração média de IGE do cacau na Saliva (p < 0,05), mas não difere significativamente da concentração média de IGE dos outros alimentos pesquisados na Saliva (p > 0,05).

- A concentração média de IGE da soja na Saliva é significativamente menor que a concentração média de IGE do leite de vaca e peixe na Saliva e maior que a concentração média de IGE do tomate e cacau na Saliva ($p < 0,05$), mas não difere significativamente da concentração média de IGE dos outros alimentos pesquisados na Saliva ($p > 0,05$).
- A concentração média de IGE do trigo na Saliva é significativamente menor que a concentração média de IGE do leite de vaca e peixe na Saliva e maior que a concentração média de IGE do tomate e cacau na Saliva ($p < 0,05$), mas não difere significativamente da concentração média de IGE dos outros alimentos pesquisados na Saliva ($p > 0,05$).
- A concentração média de IGE do trigo na Saliva é significativamente menor que a concentração média de IGE do leite de vaca e peixe na Saliva e maior que a concentração média de IGE do cacau na Saliva ($p < 0,05$), mas não difere significativamente da concentração média de IGE dos outros alimentos pesquisados na Saliva ($p > 0,05$).
- A concentração média de IGE do amendoim na Saliva é significativamente menor que a concentração média de IGE do leite de vaca e peixe na Saliva ($p < 0,05$), mas não difere significativamente da concentração média de IGE dos outros alimentos pesquisados na Saliva ($p > 0,05$).
- A concentração média de IGE do kiwi na Saliva é significativamente menor que a concentração média de IGE do leite de vaca, peixe e trigo na Saliva ($p < 0,05$), mas não difere significativamente da concentração média de IGE dos outros alimentos pesquisados na Saliva ($p > 0,05$).
- A concentração média de IGE do mamão na Saliva é significativamente menor que a concentração média de IGE do leite de vaca e peixe na Saliva e maior que a concentração média de IGE do tomate e cacau na Saliva ($p < 0,05$), mas não difere significativamente da concentração média de IGE dos outros alimentos pesquisados na Saliva ($p > 0,05$).
- A concentração média de IGE da banana na Saliva é significativamente menor que a concentração média de IGE do leite de vaca e peixe na Saliva ($p < 0,05$), mas não difere significativamente da concentração média de IGE dos outros alimentos pesquisados na Saliva ($p > 0,05$).
- A concentração média de IGE do tomate na Saliva é significativamente menor que a concentração média de IGE do leite de vaca, peixe, soja, trigo e mamão

na Saliva (p < 0,05), mas não difere significativamente da concentração média de IGE dos outros alimentos pesquisados na Saliva (p > 0,05).

- A concentração média de IGE do milho na Saliva é significativamente menor que a concentração média de IGE do leite de vaca e peixe na Saliva (p < 0,05), mas não difere significativamente da concentração média de IGE dos outros alimentos pesquisados na Saliva (p > 0,05).
- A concentração média de IGE do cacau na Saliva é significativamente menor que a concentração média de IGE do leite de vaca, peixe, ovo, soja, trigo, castanha e mamão na Saliva (p < 0,05), mas não difere significativamente da concentração média de IGE dos outros alimentos pesquisados na Saliva (p > 0,05).

Comment of table 7:

- The mean GGE concentration of cow's milk in Saliva is significantly higher than the mean GGE concentration of all the other foods investigated in Saliva (p <0.05).
- The mean GGE concentration of fish in Saliva is significantly lower than the mean GGE concentration of Saliva cow's milk (p <0.05), but it is significantly higher than the mean GGE concentration of all other foods Saliva (p <0.05).
- The mean GGE concentration of shrimp in Saliva is significantly lower than the mean GGE concentration of cow's milk and fish in Saliva (p <0.05), but does not differ significantly from the mean GGE concentration of the other foods surveyed in Saliva (p> 0.05).
- The mean GGE concentration of the egg in Saliva is significantly lower than the mean GGE concentration of cow's milk and fish in Saliva and higher than the mean GGE concentration of the cocoa in Saliva (p <0.05), but does not differ of the other foods investigated in Saliva (p> 0.05).
- The mean GGE concentration of soybean in Saliva is significantly lower than the average GGE concentration of cow's milk and fish in Saliva and higher than the mean GGE concentration of tomato and cacao in Saliva (p <0.05), but did not differ significantly from the mean GGE concentration of the other foods surveyed in Saliva (p> 0.05).
- The mean GGE concentration of wheat in Saliva is significantly lower than the mean GGE concentration of cow's milk and fish in Saliva and higher than the

mean GGE concentration of the tomato and cacao in Saliva (p <0.05), but did not differ significantly from the mean GGE concentration of the other foods surveyed in Saliva (p> 0.05).

- The mean GGE concentration of wheat in Saliva is significantly lower than the average GGE concentration of cow's milk and fish in Saliva and higher than the mean concentration of GGE in Saliva (p <0.05), but it does not differ of the other foods investigated in Saliva (p> 0.05).
- The mean GI concentration of the peanut in Saliva is significantly lower than the mean GGE concentration of cow's milk and fish in Saliva (p <0.05), but does not differ significantly from the mean GGE concentration of the other foods investigated in Saliva (p> 0.05).
- The mean concentration of KGE in Saliva is significantly lower than the average concentration of GGE in cows, fish and wheat in Saliva (p <0.05), but does not differ significantly from the average concentration of GGE in the other foods in Saliva (p> 0.05).
- The mean GGE concentration of papaya in Saliva is significantly lower than the mean GGE concentration of cow's milk and fish in Saliva and higher than the mean concentration of GGE in tomato and cacao in Saliva (p <0.05), but did not differ significantly from the mean GGE concentration of the other foods surveyed in Saliva (p> 0.05).
- The mean GI concentration of the banana in Saliva is significantly lower than the mean GGE concentration of cow's milk and fish in Saliva (p <0.05), but does not differ significantly from the average GGE concentration of the other foods surveyed in Saliva (p> 0.05).
- The mean concentration of GGE of the tomato in Saliva is significantly lower than the average concentration of GGE of cow's milk, fish, soy, wheat and papaya in Saliva (p <0.05), but does not differ significantly from the average concentration of GGE of the other foods researched in Saliva (p> 0.05).
- The mean GGE concentration of corn in Saliva is significantly lower than the mean GGE concentration of cow's milk and fish in Saliva (p <0.05), but it does not differ significantly from the mean GGE concentration of the other foods investigated in Saliva (p> 0.05).
- The mean GGE concentration of the cocoa in Saliva is significantly lower than the mean GGE concentration of cow's milk, fish, egg, soy, wheat, chestnut and

papaya in Saliva (p <0.05), but does not differ significantly from mean GGE concentration of the other foods investigated in Saliva (p> 0.05).

Quadro 8. Comparação da concentração de IGG1 no Soro entre os alimentos

Table 8. Comparison of serum IGG1 concentration between foods

Parâmetro	Alimento	n	Média	Intervalo de confiança (95%)		p£
				Limite inferior	Limite superior	
IGG1 no Soro	Leite de vaca	36	0,339 (0,048)[a]	0,323	0,355	< 0,001
	Peixe	36	0,393 (0,089)[b]	0,363	0,423	
	Camarão	36	0,203 (0,055)[c,f,g]	0,184	0,221	
	Ovo	36	0,191 (0,060)[c,d,e,f,g]	0,171	0,211	
	Soja	36	0,202 (0,060)[c,f,g]	0,181	0,222	
	Trigo	36	0,216 (0,056)[c,f]	0,197	0,235	
	Castanha	36	0,195 (0,053)[c,e,f,g]	0,177	0,212	
	Amendoim	36	0,177 (0,044)[c,e,f,g]	0,162	0,191	
	Kiwi	36	0,176 (0,045)[c,e,f,g]	0,160	0,191	
	Mamão	36	0,176 (0,050)[c,e,f,g]	0,159	0,193	
	Banana	36	0,169 (0,048)[e,g]	0,152	0,185	
	Tomate	36	0,169 (0,048)[e,g]	0,152	0,185	
	Milho	36	0,203 (0,053)[c,f,g]	0,185	0,221	
	Cacau	36	0,155 (0,032)[d,e]	0,144	0,166	

Legenda: £ - teste de Kruskal-Wallis. Letras iguais indicam que não há diferença significativa e letras diferentes indicam que há diferença significativa entre os grupos segundo o teste de Tukey. Valor de p considerado significativo menor ou igual a 0,05.

Comentário do quadro 8:

- A concentração média de IGG1 do leite de vaca no Soro é significativamente menor que a concentração média de IGG1 do peixe no Soro (p < 0,05), mas é significativamente maior que a concentração média de IGG1 de todos os outros alimentos pesquisados no Soro (p < 0,05).
- A concentração média de IGG1 do peixe no Soro é significativamente maior que a concentração média de IGG1 de todos os outros alimentos pesquisados no Soro (p < 0,05).
- A concentração média de IGG1 do camarão no Soro é significativamente menor que a concentração média de IGG1 do leite de vaca e do peixe no Soro e significativamente maior que a concentração média de IGG1 do cacau no Soro (p < 0,05), mas não difere significativamente da concentração média de IGG1 de todos os outros alimentos pesquisados no Soro (p > 0,05).

- A concentração média de IGG1 do ovo no Soro é significativamente menor que a concentração média de IGG1 do leite de vaca e do peixe no Soro ($p < 0,05$), mas não difere significativamente da concentração média de IGG1 de todos os outros alimentos pesquisados no Soro ($p > 0,05$).
- A concentração média de IGG1 da soja no Soro é significativamente menor que a concentração média de IGG1 do leite de vaca e do peixe no Soro e significativamente maior que a concentração média de IGG1 do cacau no Soro ($p < 0,05$), mas não difere significativamente da concentração média de IGG1 de todos os outros alimentos pesquisados no Soro ($p > 0,05$).
- A concentração média de IGG1 do trigo no Soro é significativamente menor que a concentração média de IGG1 do leite de vaca e do peixe no Soro e significativamente maior que a concentração média de IGG1 da banana, tomate e cacau no Soro ($p < 0,05$), mas não difere significativamente da concentração média de IGG1 de todos os outros alimentos pesquisados no Soro ($p > 0,05$).
- A concentração média de IGG1 da castanha no Soro é significativamente menor que a concentração média de IGG1 do leite de vaca e do peixe no Soro ($p < 0,05$), mas não difere significativamente da concentração média de IGG1 de todos os outros alimentos pesquisados no Soro ($p > 0,05$).
- A concentração média de IGG1 do amendoim no Soro é significativamente menor que a concentração média de IGG1 do leite de vaca e do peixe no Soro ($p < 0,05$), mas não difere significativamente da concentração média de IGG1 de todos os outros alimentos pesquisados no Soro ($p > 0,05$).
- A concentração média de IGG1 do kiwi no Soro é significativamente menor que a concentração média de IGG1 do leite de vaca e do peixe no Soro ($p < 0,05$), mas não difere significativamente da concentração média de IGG1 de todos os outros alimentos pesquisados no Soro ($p > 0,05$).
- A concentração média de IGG1 do mamão no Soro é significativamente menor que a concentração média de IGG1 do leite de vaca e do peixe no Soro ($p < 0,05$), mas não difere significativamente da concentração média de IGG1 de todos os outros alimentos pesquisados no Soro ($p > 0,05$).
- A concentração média de IGG1 da banana no Soro é significativamente menor que a concentração média de IGG1 do leite de vaca, do peixe e do trigo

no Soro (p < 0,05), mas não difere significativamente da concentração média de IGG1 de todos os outros alimentos pesquisados no Soro (p > 0,05).

- A concentração média de IGG1 do tomate no Soro é significativamente menor que a concentração média de IGG1 do leite de vaca, do peixe e do trigo no Soro (p < 0,05), mas não difere significativamente da concentração média de IGG1 de todos os outros alimentos pesquisados no Soro (p > 0,05).
- A concentração média de IGG1 do milho no Soro é significativamente menor que a concentração média de IGG1 do leite de vaca e do peixe no Soro e significativamente maior que a concentração média de IGG1 do cacau no Soro (p < 0,05), mas não difere significativamente da concentração média de IGG1 de todos os outros alimentos pesquisados no Soro (p > 0,05).
- A concentração média de IGG1 do cacau no Soro é significativamente menor que a concentração média de IGG1 do leite de vaca, do peixe, do camarão, da soja, do trigo e do milho no Soro (p < 0,05), mas não difere significativamente da concentração média de IGG1 de todos os outros alimentos pesquisados no Soro (p > 0,05).

Comment of table 8:

- The mean concentration of IGG1 in cow's milk in serum is significantly lower than the mean concentration of IGF-1 in the serum (p <0.05), but is significantly higher than the mean IGG1 concentration of all other foods Serum (p <0.05).
- The mean IGG1 concentration of the fish in serum is significantly higher than the mean IGG1 concentration of all other foods screened in serum (p <0.05).
- The mean serum IGG1 concentration of shrimp in serum is significantly lower than the mean IGG1 concentration of cow's milk and fish in serum and significantly higher than the mean concentration of IGG1 in serum (p <0.05), but does not differ significantly from the mean IGG1 concentration of all other foods screened in serum (p> 0.05).
- Mean serum IGG1 concentration in serum is significantly lower than the mean IGG1 concentration of cow's milk and fish in serum (p <0.05) but does not differ significantly from the mean IGG1 concentration of all other foods serum levels (p> 0.05).
- Mean serum IGG1 concentration in serum is significantly lower than the mean IGG1 concentration of cow's milk and fish in serum and significantly higher than

the mean IGF-1 concentration of serum cocoa (p <0.05), but does not differ significantly from the mean IGG1 concentration of all other foods screened in serum (p> 0.05).

- Mean serum IGG1 concentration in Whey is significantly lower than the mean IGG1 concentration of cow's milk and fish in Whey and significantly higher than the mean concentration of IGG1 of banana, tomato and cocoa in Whey (p <0, 05), but did not differ significantly from the mean IGG1 concentration of all other foods screened in serum (p> 0.05).

- Mean serum IGG1 concentration in serum is significantly lower than the mean IGG1 concentration of cow's milk and fish in serum (p <0.05) but does not differ significantly from the mean IGF-1 concentration of all other foods serum levels (p> 0.05).

- The mean serum IGG1 concentration of Whey Peanut is significantly lower than the mean IGG1 concentration of cow's milk and fish in serum (p <0.05), but does not differ significantly from the mean IGG1 concentration of all other foods serum levels (p> 0.05).

- The mean IGF-1 concentration of kiwi in serum is significantly lower than the mean IGG1 concentration of cow's milk and fish in serum (p <0.05), but does not differ significantly from the mean IGF-1 concentration of all other foods serum levels (p> 0.05).

- The mean concentration of IGF-1 in papaya in serum is significantly lower than the mean concentration of IGF-1 in cow's milk and fish in serum (p <0.05), but does not differ significantly from the mean IGF-1 concentration of all other foods serum levels (p> 0.05).

- The mean serum IGG1 concentration in the serum is significantly lower than the mean IGG1 concentration of cow's milk, fish and wheat in serum (p <0.05), but does not differ significantly from the mean IGG1 concentration of all the other foods tested in serum (p> 0.05).

- Mean serum IGG1 concentration in serum is significantly lower than the mean IGG1 concentration of cow's milk, fish and wheat in serum (p <0.05) but does not differ significantly from the mean IGG1 concentration of all the other foods tested in serum (p> 0.05).

- The mean serum IGG1 concentration in the serum is significantly lower than the mean IGG1 concentration of cow's milk and fish in serum and significantly

higher than the mean concentration of IGF-1 in serum ($p < 0.05$), but does not differ significantly from the mean IGG1 concentration of all other foods screened in serum ($p > 0.05$).

- The mean concentration of IGF-1 in whey is significantly lower than the mean concentration of IGG-1 in cow's milk, fish, shrimp, soybeans, wheat and corn in serum ($p < 0.05$), but not differs significantly from the mean IGG1 concentration of all other foods screened in serum ($p > 0.05$).

IGG1 in serum ($p < 0.05$), but does not differ significantly from the mean IGG1 concentration of all other foods screened in serum ($p > 0.05$).

- Mean serum IGG1 concentration in serum is significantly lower than the mean IGG1 concentration of cow's milk and fish in serum ($p < 0.05$) but does not differ significantly from the mean IGG1 concentration of all other foods serum levels ($p > 0.05$).

- Mean serum IGG1 concentration in serum is significantly lower than the mean IGG1 concentration of cow's milk and fish in serum and significantly higher than the mean IGF-1 concentration of serum cocoa ($p < 0.05$), but does not differ significantly from the mean IGG1 concentration of all other foods screened in serum ($p > 0.05$).

- Mean serum IGG1 concentration in Whey is significantly lower than the mean IGG1 concentration of cow's milk and fish in Whey and significantly higher than the mean concentration of IGG1 of banana, tomato and cocoa in Whey (p <0, 05), but did not differ significantly from the mean IGG1 concentration of all other foods screened in serum ($p > 0.05$).

Quadro 9. Comparação da concentração de IGG1 na Saliva entre os alimentos

Table 9. Comparison of IGF-1 concentration in Saliva between foods

Parâmetro	Alimento	n	Média	Intervalo de confiança (95%)		$p^{‡}$
				Limite inferior	Limite superior	
IGG1 na Saliva	Leite de vaca	36	0,333 (0,067)^{a}	0,310	0,355	< 0,001
	Peixe	36	0,391 (0,081)^{b}	0,364	0,418	
	Camarão	36	0,198 (0,042)^{c,d}	0,184	0,212	
	Ovo	36	0,182 (0,045)^{c,d}	0,167	0,198	
	Soja	36	0,201 (0,045)^{c,d}	0,186	0,216	
	Trigo	36	0,221 (0,051)^{c}	0,204	0,238	
	Castanha	36	0,204 (0,051)^{c,d}	0,187	0,221	
	Amendoim	36	0,178 (0,047)^{c,d}	0,162	0,193	
	Kiwi	36	0,169 (0,044)^{d}	0,155	0,184	
	Mamão	36	0,183 (0,076)^{c,d}	0,157	0,209	
	Banana	36	0,191 (0,049)^{c,d}	0,174	0,207	
	Tomate	36	0,197 (0,057)^{c,d}	0,178	0,216	
	Milho	36	0,186 (0,047)^{c,d}	0,170	0,202	
	Cacau	36	0,165 (0,105)^{d}	0,129	0,200	

Legenda: ‡ - teste ANOVA. Letras iguais indicam que não há diferença significativa e letras diferentes indicam que há diferença significativa entre os grupos segundo o teste de Tukey. Valor de p considerado significativo menor ou igual a 0,05.

Comentário do quadro 9:

- A concentração média de IGG1 do leite de vaca na Saliva é significativamente menor que a concentração média de IGG1 do peixe na Saliva, mas é significativamente maior que a concentração média de IGG1 de todos os outros alimentos pesquisados na Saliva ($p < 0,05$).
- A concentração média de IGG1 do peixe na Saliva é significativamente maior que a concentração média de IGG1 do leite de vaca na Saliva, mas é significativamente maior que a concentração média de IGG1 de todos os outros alimentos pesquisados na Saliva ($p < 0,05$).
- A concentração média de IGG1 do camarão na Saliva é significativamente menor que a concentração média de IGG1 do leite de vaca e peixe na Saliva ($p < 0,05$), mas não difere significativamente da concentração média de IGG1 dos outros alimentos pesquisados na Saliva ($p > 0,05$).
- A concentração média de IGG1 do ovo na Saliva é significativamente menor que a concentração média de IGG1 do leite de vaca e peixe na Saliva (p <

0,05), mas não difere significativamente da concentração média de IGG1 dos outros alimentos pesquisados na Saliva ($p > 0,05$).

- A concentração média de IGG1 da soja na Saliva é significativamente menor que a concentração média de IGG1 do leite de vaca e peixe na Saliva ($p < 0,05$), mas não difere significativamente da concentração média de IGG1 dos outros alimentos pesquisados na Saliva ($p > 0,05$).
- A concentração média de IGG1 do trigo na Saliva é significativamente menor que a concentração média de IGG1 do leite de vaca e peixe na Saliva e maior que a concentração média de IGG1 do kiwi e cacau na Saliva ($p < 0,05$), mas não difere significativamente da concentração média de IGG1 dos outros alimentos pesquisados na Saliva ($p > 0,05$).
- A concentração média de IGG1 da castanha na Saliva é significativamente menor que a concentração média de IGG1 do leite de vaca e peixe na Saliva ($p < 0,05$), mas não difere significativamente da concentração média de IGG1 dos outros alimentos pesquisados na Saliva ($p > 0,05$).
- A concentração média de IGG1 do amendoim na Saliva é significativamente menor que a concentração média de IGG1 do leite de vaca e peixe na Saliva ($p < 0,05$), mas não difere significativamente da concentração média de IGG1 dos outros alimentos pesquisados na Saliva ($p > 0,05$).
- A concentração média de IGG1 do kiwi na Saliva é significativamente menor que a concentração média de IGG1 do leite de vaca, peixe e trigo na Saliva ($p < 0,05$), mas não difere significativamente da concentração média de IGG1 dos outros alimentos pesquisados na Saliva ($p > 0,05$).
- A concentração média de IGG1 do mamão na Saliva é significativamente menor que a concentração média de IGG1 do leite de vaca e peixe na Saliva ($p < 0,05$), mas não difere significativamente da concentração média de IGG1 dos outros alimentos pesquisados na Saliva ($p > 0,05$).
- A concentração média de IGG1 da banana na Saliva é significativamente menor que a concentração média de IGG1 do leite de vaca e peixe na Saliva ($p < 0,05$), mas não difere significativamente da concentração média de IGG1 dos outros alimentos pesquisados na Saliva ($p > 0,05$).
- A concentração média de IGG1 do tomate na Saliva é significativamente menor que a concentração média de IGG1 do leite de vaca e peixe na Saliva (p

< 0,05), mas não difere significativamente da concentração média de IGG1 dos outros alimentos pesquisados na Saliva (p > 0,05).
- A concentração média de IGG1 do milho na Saliva é significativamente menor que a concentração média de IGG1 do leite de vaca e peixe na Saliva (p < 0,05), mas não difere significativamente da concentração média de IGG1 dos outros alimentos pesquisados na Saliva (p > 0,05).
- A concentração média de IGG1 do cacau na Saliva é significativamente menor que a concentração média de IGG1 do leite de vaca, peixe e trigo na Saliva (p < 0,05), mas não difere significativamente da concentração média de IGG1 dos outros alimentos pesquisados na Saliva (p > 0,05).

Comment of table 9:

-The mean IGG1 concentration of cow's milk in Saliva is significantly lower than the mean IGG1 concentration of the fish in Saliva, but it is significantly higher than the mean IGG1 concentration of all the other foods surveyed in Saliva (p <0.05).
-The mean IGG1 concentration of the fish in Saliva is significantly higher than the mean IGG1 concentration of cow's milk in Saliva, but it is significantly higher than the mean IGG1 concentration of all other foods surveyed in Saliva (p<0.05).
- The mean IGG1 concentration of shrimp in Saliva is significantly lower than the mean IGG1 concentration of cow's milk and fish in Saliva (p <0.05), but does not differ significantly from the mean IGG1 concentration of the other foods surveyed in Saliva (p> 0.05).
- The mean IGG1 concentration of the egg in Saliva is significantly lower than the mean IGG1 concentration of cow's milk and fish in Saliva (p <0.05), but does not differ significantly from the mean IGG1 concentration of the other foods surveyed in Saliva (p> 0.05).
- The mean IGG1 concentration of soybean in Saliva is significantly lower than the mean IGG1 concentration of cow's milk and fish in Saliva (p <0.05), but does not differ significantly from the mean IGG1 concentration of the other foods surveyed in Saliva (p> 0.05).
- The mean IGG1 concentration of wheat in Saliva is significantly lower than the mean IGG1 concentration of cow's milk and fish in Saliva and higher than the

mean IGG1 concentration of kiwi and cacao in Saliva ($p < 0.05$), but does not differ significantly from the mean IGG1 concentration of the other foods surveyed in Saliva ($p > 0.05$).

- The mean IGG1 concentration of the chestnut in Saliva is significantly lower than the mean IGG1 concentration of cow's milk and fish in Saliva ($p < 0.05$), but does not differ significantly from the mean IGG1 concentration of the other foods surveyed in Saliva ($p > 0.05$).
- The mean IGG1 concentration of peanut in Saliva is significantly lower than the mean IGG1 concentration of cow's milk and fish in Saliva ($p < 0.05$), but does not differ significantly from the mean IGG1 concentration of the other foods surveyed in Saliva ($p > 0.05$).
- The mean IGI concentration of kiwifruit in Saliva is significantly lower than the mean IGG1 concentration of cow, fish and wheat milk in Saliva ($p < 0.05$), but does not differ significantly from the mean IGG1 concentration of the other foods in Saliva ($p > 0.05$).
- The mean IGG1 concentration of papaya in Saliva is significantly lower than the mean IGG1 concentration of cow's milk and fish in Saliva ($p < 0.05$), but does not differ significantly from the mean IGG1 concentration of the other foods surveyed in Saliva ($p > 0.05$).
- The mean IGG1 concentration of banana in Saliva is significantly lower than the mean IGG1 concentration of cow's milk and fish in Saliva ($p < 0.05$), but does not differ significantly from the mean IGG1 concentration of the other foods surveyed in Saliva ($p > 0.05$).
- The mean IGG1 concentration of tomato in Saliva is significantly lower than the mean IGG1 concentration of cow's milk and fish in Saliva ($p < 0.05$), but does not differ significantly from the mean IGG1 concentration of the other foods researched in Saliva ($p > 0.05$).
- The mean IGG1 concentration of corn in Saliva is significantly lower than the mean IGG1 concentration of cow's milk and fish in Saliva ($p < 0.05$), but does not differ significantly from the mean IGG1 concentration of the other foods surveyed in Saliva ($p > 0.05$).
- The mean IGG1 concentration of cocoa in Saliva is significantly lower than the mean IGG1 concentration of cow, fish and wheat milk in Saliva ($p < 0.05$), but

does not differ significantly from the mean IGG1 concentration of the other foods in Saliva (p> 0.05).

Figura 1. Concentração de IGE no soro, IGE na saliva, IGG1 no soro e IGG1 na saliva dos 14 alimentos pesquisados

Figure 1. Concentration of GGE in serum, GGE in saliva, serum IGG1 and IGG1 in the saliva of the 14 foods researched

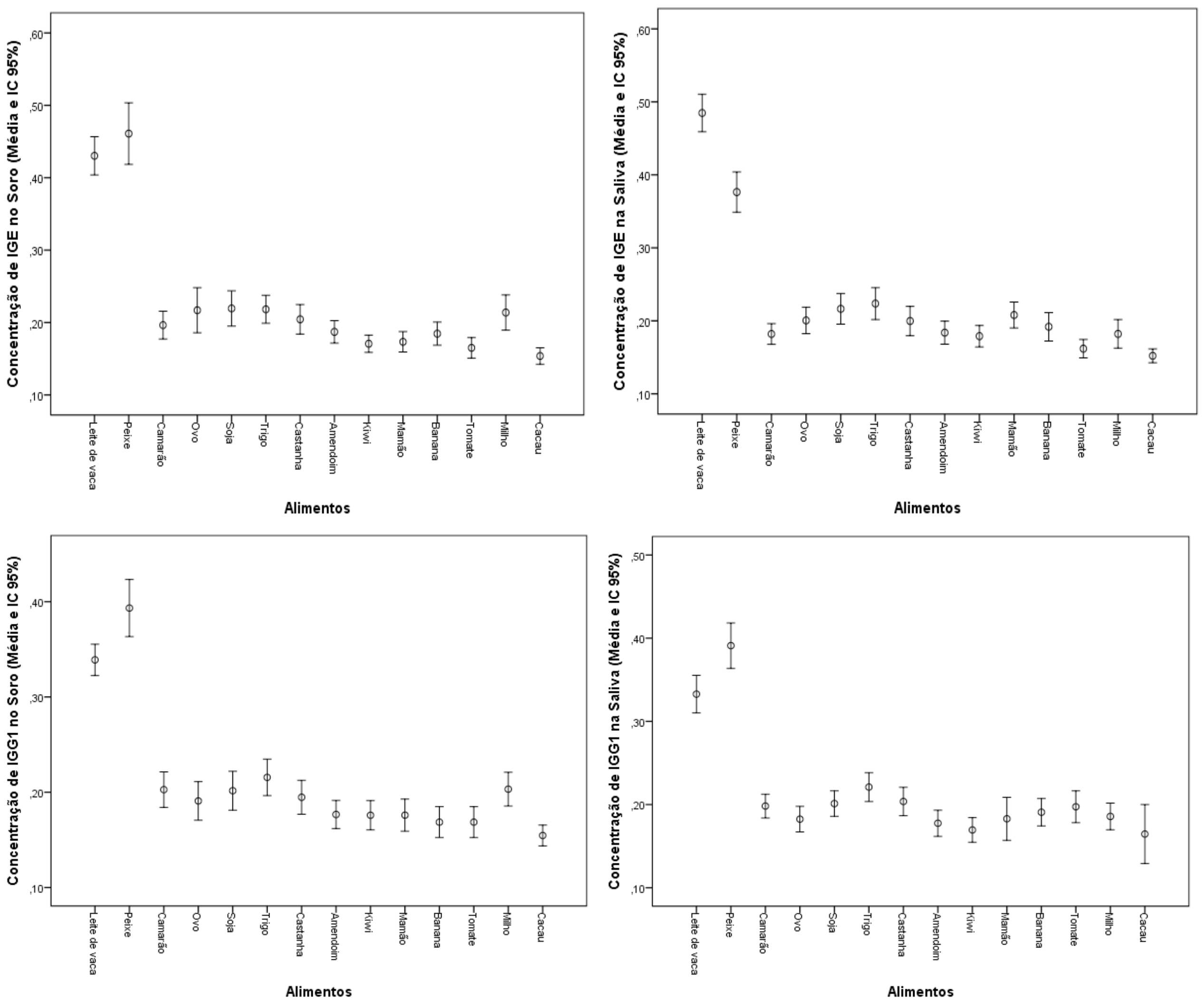

Comentário da figura 1:

Na figura 1 acima estão apresentados os gráficos de barras de erros com a média e intervalo de confiança de 95% das concentrações de IGE no soro, IGE na saliva, IGG1 no soro e IGG1 na saliva para cada um dos alimentos pesquisados.

Os valores de média e os intervalos de confiança de 95% das variáveis IGE no soro, IGE na saliva, IGG1 no soro e IGG1 na saliva estão descritos nos quadros 6, 7, 8 e 9, respectivamente.

Para o melhor entendimento de como poderão ser interpretadas as barras dos gráficos, será colocada abaixo a interpretação da barra referente a concentração de IGE no soro, IGE na saliva, IGG1 no soro e IGG1 na saliva do leite de vaca.

Com uma confiança de 95%, a média da concentração de IGE do leite de vaca no soro é 0,430, e se encontra dentro do intervalo de 0,404 a 0,457. Com uma confiança de 95%, a média da concentração de IGE do leite de vaca na saliva é 0,485, e se encontra dentro do intervalo de 0,459 a 0,510. Com uma confiança de 95%, a média da concentração de IGG1 do leite de vaca no soro é 0,339, e se encontra dentro do intervalo de 0,323 a 0,355. Com uma confiança de 95%, a média da concentração de IGG1 do leite de vaca na saliva é 0,333, e se encontra dentro do intervalo de 0,310 a 0,355.

Comment of figure 1:

In figure 1 above the bar graphs of errors with the mean and 95% confidence interval of the concentrations of serum GGE, salivary GI, serum IGG1 and IGG1 in saliva for each of the foods investigated are presented.

The mean values and the 95% confidence intervals of the serum GGE, GGE in saliva, serum IgG1 and IGG1 in saliva are described in Tables 6, 7, 8 and 9, respectively.

For a better understanding of how the bars of the graphs can be interpreted, the interpretation of the bar referring to the concentration of GGE in the serum, IGE in the saliva, IGG1 in the serum and IGG1 in the saliva of the cow's milk will be placed below.

At 95% confidence, the mean serum GI concentration of cow's milk is 0.430, and is within the range of from 0.404 to 0.457. At 95% confidence, the mean GI concentration of cow's milk in saliva is 0.485, and is in the range of 0.459 to 0.510. At a 95% confidence level, the mean IGG1 concentration of cow's milk in serum is 0.339, and is in the range of 0.323 to 0.355. At a 95% confidence level, the mean IGG1 concentration of cow's milk in saliva is 0.333, and is within the range of 0.310 to 0.355.
