# Supplementary material for: Detection of serum and salivary IgE and IgG1 immunoglobulins specific for diagnosis of food allergy
Source: PLoS One. 2019 Apr 17;14(4):e0214745. doi: 10.1371/journal.pone.0214745 (PMC6469776; doi:10.1371/journal.pone.0214745)
Supplement: S3 File — (PDF) [file pone.0214745.s003.pdf]

TERMO DE CONSENTIMENTO LIVRE ESCLARECIDO
Universidade Estadual do Ceará – UECE
Centro de Ciências da Saúde – CCS
Mestrado em Nutrição e Saúde

ESTAMOS CONVIDANDO SEU/SUA FILHO (A) PARA PARTICIPAR DA PESQUISA intitulada**: PADRONIZAÇÃO DE TESTE DIAGNÓSTICO PARA ALERGIA ALIMENTAR EM CRIANÇAS. Esclareço que:** As informações coletadas somente serão utilizadas para os objetivos da pesquisa. O Senhor ou a Senhora tem a liberdade de desistir a qualquer momento de permitir que seu filho participe da pesquisa. Esclarecemos também, que as informações ficarão em sigilo e que seu anonimato será preservado. Em nenhum momento o senhor (a) ou seu filho terão prejuízo no tratamento e /ou financeiro. Informamos, que a participação da pesquisa não implica em riscos podendo o (a) Sr. (a) recusar-se a participar sem nenhum tipo de penalização ou qualquer prejuízo.

Objetivo Geral: Padronizar uma técnica sorológica para diagnostico de reações alérgicas utilizando o soro e saliva. **Metodologia:** Serão coletados 3mL de sangue e colocado em tubo sem heparina, e também de saliva (quantidade existente no Salivet). No laboratório de análise da UECE será centrifugada e armazenada em temperatura adequada (- 40º C). Os antissoros dos pacientes serão submetidos a teste de ELISA indireto (Enzyme Linked Immunosorbent Assay) para detecção de IgE e IgG1. Para esse ensaio, as placas serão sensibilizadas com diferentes alimentos (3µg/orifício). Os antígenos (alimentos) serão diluídos em tampão de carbonato de sódio 50 mM, pH 9,6, sendo usado 100 µl em cada poço. As placas serão incubadas 'overnight' a uma temperatura de 4°C, e em seguida, o soro de cada paciente suspeito de reações alérgicas será utilizado como anticorpo primário. **Benefícios:** As crianças participantes pesquisa, serão beneficiados pela análise de quais alimentos estão propensas a desencadear reações alérgicas. **Riscos e desconfortos esperados:** O risco e desconforto gerado pela pesquisa será a coleta de sangue, porém será minimizado por aproveitar o mesmo procedimento de rotina solicitado pelas gastropediatras do ambulatório bem como sangue ser coletado por enfermeira do próprio hospital. Os pesquisadores não identificam qualquer outro risco para seu filho e sua família. Importante dizer que a pesquisa atenderá a resolução 466/12 II.2.3 do Conselho Nacional de Saúde de 12 de Dezembro e 2012, a qual garante assistência integral as complicações e danos decorrentes, direta ou indiretamente, da pesquisa. **Acesso aos resultados:** Todo participante da pesquisa poderá ter acesso periódico aos resultados relevantes obtidos, pois após a conclusão da dissertação a ser elaborado com os dados obtidos, o mesmo será submetido a direção do hospital para aprovação e estará a disposição das famílias das crianças participantes. A pesquisa seguirá todas as normas e diretrizes da resolução 466 do Conselho Nacional de Saúde de 12 de Dezembro e 2012.

Se houver dúvidas sobre a ética da pesquisa entre em contato com o Comitê de Ética em pesquisa do Hospital Albert Sabin, Rua Tertuliano Sales, 544 – Vila União - CEP: 60410-790, Fortaleza – Ceará ou Fone: (85) 3101.4200.

Sendo necessário contatar com os pesquisadores responsáveis escreva ou telefone para:

Pesquisador: Maria Izabel Florindo Guedes. Telefone: (85) 8807.4003
Pesquisador: Marília Porto Oliveira Telefone: (85) 8818.1221

Gostaríamos de colocar que sua participação será de extrema importância para esse trabalho. Dados do entrevistado:

Nome:_______________________________________**RG:**________________________

Endereço:____________________________________**Tel:**_______________________

-- --
Representante Legal Pesquisador

TRANSLATION

CLOSED FREE CONSENT TERM
State University of Ceará - UECE
Health Sciences Center - CCS
Master in Nutrition and Health

WE ARE INVITING YOUR / YOUR CHILD TO PARTICIPATE IN THE RESEARCH entitled: DIAGNOSTIC TEST STANDARDS FOR FOOD ALLERGY IN CHILDREN. I clarify that: The information collected will only be used for the purposes of the research. You are free to give up at any time to allow your child to participate in the survey. We also clarify that the information will be kept confidential and that its anonymity will be preserved. At no time will you or your child be injured in the treatment and / or financial. We inform you that the participation of the research does not imply risks and you can refuse to participate without any kind of penalty or any loss.
Objective: To standardize a serological technique for the diagnosis of allergic reactions using serum and saliva. Methodology: 3mL of blood will be collected and placed in a tube without heparin, and also saliva (Salivet amount). In the analysis laboratory of the UECE will be centrifuged and stored at a suitable temperature (- 40 ° C). Patients' antisera will be submitted to indirect ELISA (Enzyme Linked Immunosorbent Assay) for IgE and IgG1 detection. For this assay, the plates will be sensitized with different foods (3µg / well). The antigens (food) will be diluted in 50 mM sodium carbonate buffer, pH 9.6, using 100 µl in each well. The plates will be incubated overnight at a temperature of 4 ° C, and then the serum of each patient suspected of allergic reactions will be used as the primary antibody. Benefits: The children participating in research will benefit from the analysis of which foods are prone to trigger allergic reactions. Risks and discomforts expected: The risk and discomfort generated by the research will be the collection of blood, but will be minimized by taking advantage of the same routine procedure requested by the ambulatory gastropediatricians as well as blood being collected by the hospital's own nurse. Researchers do not identify any other risk to their child and family. It is important to state that the research will comply with Resolution 466/12 II.2.3 of the National Health Council of December 12, 2012, which guarantees full assistance for complications and damages arising directly or indirectly from the research. Access to the results: Every research participant may have periodic access to the relevant results obtained, because after the conclusion of the dissertation to be elaborated with the data obtained, it will be submitted to the hospital management for approval and will be available to the families of the participating children . The research will follow all the guidelines and guidelines of Resolution 466 of the National Health Council of December 12 and 2012.

If there are doubts about the research ethics, contact the Research Ethics Committee of the Albert Sabin Hospital, Rua Tertuliano Sales, 544 - Vila União - CEP: 60410-790, Fortaleza - Ceará or Phone: (85) 3101.4200.

If you need to contact the responsible researchers write or call:
Researcher: Maria Izabel Florindo Guedes. Phone: (+5585) 8807.4003
Researcher: Marília Porto Oliveira Phone: (+5585) 8818.1221

We would like to point out that your participation will be extremely important for this work. Data of the interviewee:

Name: ________________________________ ID: ________________________

Address: ________________________________Phone: ___________________

------------------------ Legal Representative

------------------------- Researcher
