# Supplementary material for: Detection of serum and salivary IgE and IgG1 immunoglobulins specific for diagnosis of food allergy
Source: PLoS One. 2019 Apr 17;14(4):e0214745. doi: 10.1371/journal.pone.0214745 (PMC6469776; doi:10.1371/journal.pone.0214745)
Supplement: S5 File — (DOCX) [file pone.0214745.s005.docx]

**SUPPLEMENTARY MATERIAL 1**

**EXAMINATION OF FOOD HYPERSENSITIVITY**

**NAME:______________________________________READER:__________**

**DATE OF BIRTH:__________________CURRENT AGE:__________________**

**MOTHER:_____________________________________________________________**

**FATHER:_____________________________________________________________**

**PHONE:_______________________________DATE:______/_______/_______**

**BREASTFEEDING: ( ) YES, EXCLUSIVE UNTIL___________**

**MISTA UNTIL:_____________________**

**( )NO**

**MAIN COMPLAINT:................................**

**CURRENT DISEASE HISTORY**

**____________________________________________________________________________________________________________________________________________**

**MARCAR COM UM X...........................**

| **DISEASE** | **AGE** | **UNLEASHING** | **REATMENT OU DIET** |
| --- | --- | --- | --- |
| **URTICARIA** |  |  |  |
| **ANGIODEMA** |  |  |  |
| **DERMATITIS** |  |  |  |
| **VOMITING** |  |  |  |
| **DIARRHEA** |  |  |  |
| **ABDOMINAL PAIN** |  |  |  |
| **LOW WEIGHT GAIN** |  |  |  |
| **BLEEDINGS** |  |  |  |
| **LARINGITES** |  |  |  |
| **OTHERS** |  |  |  |

**CO-MORBIDITIES:**

**__________________________________________________________________________________________________________________________________________________________________________________________________________________**

**OTHER ALLERGIES:**

**__________________________________________________________________________________________________________________________________________________________________________________________________________________**

**FAMILY BACKGROUND:**

**MOTHER:____________________________________________________________**

**FATHER:____________________________________________________________**

**BROTHERS:___________________________________________________________**

**FOOD HISTORY**

**INTRODUCTION OF SOLIDS:_________________________________________**

**CURRENT DIET:**

**BREAKFAST:_________________________________________________________SNACK:_____________________________________________________________ LUNCH:____________________________________________________________ SNACK:_____________________________________________________________**

**DINNER:______________________________________________________________ OTHERS:_____________________________________________________________**

**PHYSICAL EXAM**

**WEIGHT:________________________PERCENTILE:_______________________STATURE:______________________PERCENTILE:________________________TURGOR:_____________________________________________________________**

**CUTANEOUS DPUBLES:____________________________________________________________**

**CIRCUNFERENCES:___________________________________________________**

**LABORATORY EVALUUATION:**

**IgE:**

**IgA:**

**Ac Anti-transglutaminase:**

**Ac Anti-gliadina:**

**Ac Anti-endomisio:**

**Rast para alimentos ou PICK:**

**Alfa-1-antitripsina:**

**Bx com contagem de eosinófilos:**

**Bx com contagem de eosinófilos:**

**EDA:**

**COLONOSCOPY:**

**PHMETRIA:**

**OTHERS:**

**CONDUCT:__________________________________________________________________________________________________________________________________**

**RETURN DATE: __________________________**
